# Feature selection and causal analysis for microbiome studies in the presence of confounding using standardization

**DOI:** 10.1186/s12859-021-04232-2

**Published:** 2021-07-06

**Authors:** Emily Goren, Chong Wang, Zhulin He, Amy M. Sheflin, Dawn Chiniquy, Jessica E. Prenni, Susannah Tringe, Daniel P. Schachtman, Peng Liu

**Affiliations:** 1 Department of Statistics, Iowa State University, 2438 Osborn Dr, Ames, IA 50011 USA; 2 Department of Veterinary Diagnostic and Production Animal Medicine, Iowa State University, 2203 Lloyd Veterinary Medical Center, Ames, IA 50011 USA; 3 Department of Horticulture and Landscape Architecture, Colorado State University, 301 University Ave, Fort Collins, CO 80523 USA; 4 Department of Energy, Joint Genome Institute, 2800 Mitchell Dr, Walnut Creek, CA 94598 USA; 5 Department of Agronomy and Horticulture, University of Nebraska, 1825 N 38th St, Lincoln, NE 68583 USA

**Keywords:** High-dimensional feature selection, Microbiome analysis, Next-generation sequencing, Standardization, Causal inference

## Abstract

**Background:**

Microbiome studies have uncovered associations between microbes and human, animal, and plant health outcomes. This has led to an interest in developing microbial interventions for treatment of disease and optimization of crop yields which requires identification of microbiome features that impact the outcome in the population of interest. That task is challenging because of the high dimensionality of microbiome data and the confounding that results from the complex and dynamic interactions among host, environment, and microbiome. In the presence of such confounding, variable selection and estimation procedures may have unsatisfactory performance in identifying microbial features with an effect on the outcome.

**Results:**

In this manuscript, we aim to estimate population-level effects of individual microbiome features while controlling for confounding by a categorical variable. Due to the high dimensionality and confounding-induced correlation between features, we propose feature screening, selection, and estimation conditional on each stratum of the confounder followed by a standardization approach to estimation of population-level effects of individual features. Comprehensive simulation studies demonstrate the advantages of our approach in recovering relevant features. Utilizing a potential-outcomes framework, we outline assumptions required to ascribe causal, rather than associational, interpretations to the identified microbiome effects. We conducted an agricultural study of the rhizosphere microbiome of sorghum in which nitrogen fertilizer application is a confounding variable. In this study, the proposed approach identified microbial taxa that are consistent with biological understanding of potential plant-microbe interactions.

**Conclusions:**

Standardization enables more accurate identification of individual microbiome features with an effect on the outcome of interest compared to other variable selection and estimation procedures when there is confounding by a categorical variable.

**Supplementary Information:**

The online version contains supplementary material available at 10.1186/s12859-021-04232-2.

## Introduction

Advancements in next-generation sequencing (NGS) technologies have recently allowed for unprecedented examination of the community of microorganisms in a host or site of interest, referred to as a microbiome [29]. Early cultivation-dependent methods only allowed for detection of a small fraction of the total microbial species present. In contrast, NGS technologies can rapidly detect thousands of microbes in each sample by determining the nucleotide sequences of short microbial DNA fragments. These fragments may either correspond to targets of a specific genetic marker, commonly the 16S ribosomal RNA gene for taxonomic identification of bacteria as in amplicon sequencing, or result from shearing all the DNA in a sample as in shotgun metagenome sequencing [40]. For each fragment, the corresponding nucleotide sequence is referred to as a “read,” the length of which is dependent on the specific NGS system [33].

Both amplicon-based and shotgun metagenomic approaches can enumerate the relative abundance of thousands of microbial features per sample. Use of amplicon sequencing for microbial enumeration is more common than shotgun metagenome sequencing due to reduced cost and complexity. For this reason, we focus on amplicon-based microbiome data here, and refer the reader to Sharpton [47] for detailed coverage of metagenomic sequencing and Knight et al. [28] for a thorough comparison of the two approaches. In order to enumerate microbes, amplicon reads are typically clustered into operational taxonomic units (OTUs) according to a fixed level of sequence similarity (e.g., 97%) [62], or as advocated by Callahan et al. [8], enumerated on the basis of denoised sequences termed exact amplicon sequence variants (ASVs). Both OTUs and ASVs may be classified into known taxa [44]. The resulting microbiome data for each sample are high-dimensional nonnegative integer counts across potentially thousands of features (taxa, OTUs, or ASVs). These counts represent relative, not absolute, numbers for each sample due to varying library sizes, a technical limitation of NGS approaches. Consequently, microbiome data must be normalized, rarefied, or treated as compositional in order to make comparisons across samples and it is unresolved which method is optimal for a particular research question and data set [17, 35, 61].

Microbiome studies have uncovered associations between microbes and human, animal, and plant health outcomes. Randomized clinical trials have been performed to determine the causal effect of fecal microbiota transplantation [9], but these do not provide causal inference on the contribution of individual microbiome features. It is important to identify individual microbiome features with a causal effect on the outcome because such discoveries may lead to development of microbial interventions for treatment of disease or optimization of crop yields. A recent review highlights the importance of identifying individual taxa with biologically relevant roles in microbiome studies [3].

Recently, there has been interest in causal inference in microbiome studies [65]. The gold standard for causal inference is to randomly assign treatments (here, microbiome interventions) and estimate the causal effect. However, this is challenging in microbiome studies since many microorganisms cannot be directly cultured [53], and random assignment of microbiomes to units is often not possible. To date, causal inference in microbiome studies has been primarily limited to causal mediation analysis that determines if a causal effect of treatment is transmitted through the microbiome [52, 58, 71]. Software has been developed to apply Granger causality [19] to microbiome time series [2], but the performance of such an approach has not been thoroughly evaluated using simulation studies.

In this work, we aim to identify individual microbial features with a causal effect on an outcome in a population of interest using causal inference. Here, the microbiome features are considered to be multivariate exposures, and are often of much higher dimension than the sample size. Previous work on high-dimensional causal inference is typically limited to settings with high-dimensional confounders rather than exposures (e.g., Schneeweiss et al. [45]) or directed graphical modeling [38]. Recently, Nandy et al. [36] considered directed graphical modeling for estimation of joint simultaneous interventions. However, their approach requires linearity and Gaussianity assumptions for high-dimensional inference, which are inappropriate for microbiome count data. There are proposed approaches for causal inference for multivariate exposures or treatments using the potential-outcomes framework, and such approaches often rely on the generalized propensity score [24]. Siddique et al. [50] compared inverse probability of treatment weighting, propensity score adjustment, and targeted maximum likelihood approaches for multivariate exposures. Wilson et al. [64] proposed Bayesian model averaging over different sets of confounders when the set of true confounding variables is unknown. When the exposures are time-varying, Taubman et al. [54] considered *g*-estimation and Hernán et al. [21] proposed a marginal structural model. However, in all of these studies with multivariate exposures, the exposure dimensionality is smaller than the sample size.

In addition to the high dimensionality, causal inference for microbiome studies is complicated by potentially complex interactions among host, environment, and microbiome. For example, there could be categorical confounding variables that affect both the outcome and some of the microbiome features. To overcome the challenges of the high dimensionality and presence of categorical confounding variables in microbiome studies, we propose standardization on the confounder and use the potential-outcomes framework for causal inference [26]. The potential-outcomes framework [22, 37, 42] conceptually frames causal inference as a missing data problem: the outcome can only be measured under the exposure actually received, making the outcome unobservable under all other possible values of the exposure. We refer the reader to Hernán and Robins [20] for a more detailed introduction. To deal with high-dimensionality of the microbiome exposure and categorical confounding variables, we propose variable screening, selection, and estimation of microbiome effects conditional on the confounder (i.e., stratification), followed by standardization to obtain estimates of effects in the population of interest. Conditioning on the confounder for microbiome feature screening, selection, and estimation avoids complications due to high marginal confounder-induced correlation between features. Further, conditional estimation naturally allows for effect modification (i.e., interaction between the confounder and microbiome features), affording flexibility to capture host-environment-microbiome interactions. Standardization allows for estimation and ranking of microbiome feature effects in the target population, which has policy and epidemiological relevance. Even if conditions for causal inference do not hold, avoiding such marginal correlation allows for superior identification of associational microbiome effects.

In this manuscript we begin by defining the estimands of interest and outlining conditions required for causal inference in "Model and assumptions" section. We then propose our estimation approach with standardization in "Methods" section. Next, we demonstrate the feasibility of our approach through simulation studies in "Simulation studies" section and present a real data application using an agricultural microbiome study in "Real data analysis" section. This paper ends with a discussion and conclusion.

## Model and assumptions

### Notation, microbiome effects, confounding

Consider a study with *n* samples (indexed by $i=1,\dots ,n$) aimed at identifying the population effect ${\varvec{\beta }} = (\beta _1, \dots , \beta _p)'$ of *p* microbiome features (e.g., taxa, ASVs, OTUs) ${\varvec{A}}_i = (A_{i1}, \dots , A_{ip})'$ on an outcome $Y_i \in {\mathbb {R}}$, such as a health response of interest. For formulating the estimand, we assume that ${\varvec{A}}_i$ has been appropriately normalized. Importantly, $Y_i$ represents the observed outcome for sample *i*, which differs from the notion of a potential outcome [42]. Define the potential outcome $Y_i^{\varvec{a}}$ as the value the outcome would take under the (possibly counterfactual) microbiome value ${\varvec{a}} = (a_1, \dots , a_p)'$. Assume that the expected potential outcome is related to the population effect ${\varvec{\beta }}$ through a linear function of the microbiome features as

1 $$\begin{aligned} {{\,\mathrm{E}\,}}\left( Y_i^{\varvec{a}}\right) = \beta _0 + \sum _{j=1}^p \beta _j a_j, \end{aligned}$$

where for each *j*, $\beta _j$ represents the effect of the *j*th microbiome feature in the population and $a_j$ is the potential or counterfactual value of the *j*th microbiome feature. In terms of (1), identifying which microbiome features have a causal effect on the response corresponds to estimation and inference for $\beta _j$ ($1\le j\le p)$. For generality, the formulation of  (1) ignores possible microbe-microbe interactions and any constraints of carrying capacity.

Note that the model in (1) is defined for the potential outcomes, not the observed data, and is thus a marginal structural model [21]. In the presence of a confounding variable $L_i$ that affects both ${\varvec{A}}_i$ and $Y_i$, this model generally does not hold for the observed data because confounding implies ${{\,\mathrm{E}\,}}\left( Y_i^{\varvec{a}}\right) \ne {{\,\mathrm{E}\,}}\left( Y_i \,\vert \,{\varvec{A}}_i = {\varvec{a}} \right)$. Consequently, specific assumptions and methodology are required to obtain an estimator $\hat{\varvec{\beta }}$ of ${\varvec{\beta }}$ that has causal, rather than merely associational, interpretation. In the next sub-section, we address the assumptions required for such a causal interpretation. We restrict our attention to the case where the confounder $L_i$ is categorical with a finite number of levels, each represented sufficiently in the study of *n* samples.

### Assumptions for causal inference

Under the potential-outcomes framework, ascribing a causal interpretation to an estimate of ${\varvec{\beta }}$ requires three assumptions: positivity, conditional exchangeability, and consistency [20]. Positivity requires positive probability for each possible microbiome level, conditional on the confounder. To formalize this, let ${\mathcal {A}}$ denote the set of all possible microbiome values in the population. The positivity condition holds if ${{\,\mathrm{Pr}\,}}\left( {\varvec{A}}_i = {\varvec{a}} \,\vert \,L_i = l\right) > 0$ for all ${\varvec{a}} \in {\mathcal {A}}$ and all levels *l* of confounder $L_i$ such that ${{\,\mathrm{Pr}\,}}(L_i = l) \ne 0$ in the population of interest, henceforth denoted by the set ${\mathcal {L}}$. Clearly, if a given microbe is either absent or below the limit of detection across all samples, its effect on the response cannot be determined. Hence, this assumption requires a large enough sequencing depth in order to sufficiently enumerate any present microbes with a causal effect. Practical considerations for evaluating the positivity assumption are covered by Westreich and Cole [63].

To meet the conditional exchangeability requirement, the data-generating mechanism for each possible microbiome must depend only on the confounder, formalized as  for all $l \in {\mathcal {L}}$, where  denotes statistical independence. Conditional exchangeability requires no unmeasured confounding. This assumption is most justifiable in experiments where the confounder is randomly assigned as in our motivating study described later in "Real data analysis" section, where agricultural plots are randomized to either low or high nitrogen fertilizer.

The consistency criterion is met if the observed outcome for each unit is the potential outcome under the observed microbiome, formally stated as ${\varvec{A}}_i = {\varvec{a}} \implies Y_i^{\varvec{a}} = Y_i$. For microbiome data, this necessitates appropriate normalization. Since NGS-based technologies enumerate based on genetic material, the resulting counts can arise from both viable and non-viable microbes [6]. In order to met the consistency assumption, relevant microbes with the same normalized count cannot have disparate effects due to differential viability. When there is concern that this assumption may be violated, it is possible to restrict amplification of RNA target genes to only viable bacterial cells [41]. We note that even if these three conditions cannot be verified, our proposed method has utility in estimation of associational, rather than causal, effects.

## Methods

### Standardization

Our goal is to estimate the population microbiome effects ${\varvec{\beta }}$ of (1) and infer which microbiome features are relevant to the response, that is, $\{1 \le j \le p: \beta _j \ne 0 \}$. We propose computing an estimate $\hat{\varvec{\beta }}^l$ for each stratum $l \in {\mathcal {L}}$ of the confounder, followed by standardization to the confounder distribution, thereby obtaining a population-level estimate $\hat{\varvec{\beta }}$. Under the assumptions stated in "Assumptions for causal inference" section, there is no confounding within each stratum *l* of the confounder. Beyond elimination of confounding, conditioning on a stratum of the confounder avoids marginal correlation between features induced by the relationship with the confounder that can hinder feature selection performance. Figure S7 in the Additional file 1 shows microbiome data from an agricultural study described in "Real data analysis" section where many features are highly correlated when considered marginally, but are relatively uncorrelated within each level of a fertilizer confounder. Combining the assumptions of "Assumptions for causal inference" section with the model in (1) and allowing for effect modification, we have

2 $$\begin{aligned} {{\,\mathrm{E}\,}}\left( Y_i \,\vert \,{\varvec{A}}_i = {\varvec{a}}, L_i = l \right) = \beta _0^l + \sum _{j=1}^p \beta _j^l a_j, \end{aligned}$$

where ${\varvec{\beta }}^l = (\beta _1^l, \dots , \beta _p^l)'$ is the corresponding stratum-specific effect. There is effect modification if ${\varvec{\beta }}^l \ne {\varvec{\beta }}^{l'}$ for some $l \ne l' \in {\mathcal {L}}$.

Standardizing the stratum-specific mean outcomes to the confounder distribution produces the population mean outcome function

3 $$\begin{aligned} {{\,\mathrm{E}\,}}\left( Y_i \,\vert \,{\varvec{A}}_i\right) = \sum _{l \in {\mathcal {L}} } \left( \beta _0^l + \sum _{j=1}^p A_{ij} \beta _j^l\right) {{\,\mathrm{Pr}\,}}\left( L_i = l\right) . \end{aligned}$$

By linearity, the effect in the population corresponding to a one-unit increase in the *j*th microbiome feature, controlling for all others, is represented by $\beta _j = \sum _{l\in {\mathcal {L}}}\beta _j^l {{\,\mathrm{Pr}\,}}\left( L_i = l\right)$ for $j = 1, \dots , p$. Given a suitable estimator $\hat{\varvec{\beta }}^l$ of ${\varvec{\beta }}^l$ for all $l \in {\mathcal {L}}$, the resulting population-standardized estimate of $\beta _j$ is

4 $$\begin{aligned} {\hat{\beta }}_j = \sum _{l\in {\mathcal {L}}}\hat{\beta _j^l} {{\,\mathrm{Pr}\,}}\left( L_i = l\right) . \end{aligned}$$

Essentially, each stratum-specific estimate is weighted by the prevalence of the confounder in the target population, represented by ${{\,\mathrm{Pr}\,}}\left( L_i = l\right)$. The population-level value is obtained through a weighted average of stratum-specific estimates.

### Feature selection and estimation

In this section, we propose a feature selection and estimation procedure for stratum-specific coefficients ${\varvec{\beta }}^l$, performed independently for each confounder level $l \in {\mathcal {L}}$. Within each stratum, we make a sparsity assumption that few microbiome features have an effect on the response and correspondingly most entries of ${\varvec{\beta }}^l$ are zero, and also assume that the outcome is normally distributed with constant variance. Commonly, $n \ll p$ for microbiome features for taxa at the level of species (and perhaps genera), OTUs, or ASVs. Consequently, we suggest penalized least squares estimation that induces shrinkage towards zero via a penalty function $p_{\lambda }$, where $\lambda$ is a tuning parameter controlling the amount of shrinkage. We suggest choosing $\lambda$ using the Bayesian information criterion (BIC) [46] due to its consistency property in selecting the true features in certain settings [59] and nonconsistency of prediction accuracy criteria such as cross-validation [30]. Possible choices for penalties that perform variable selection through shrinkage-induced sparsity include the least absolute shrinkage and selection operator (LASSO) [55] and smoothly clipped absolute deviation (SCAD) [13], among others [69].

Due to the high dimensionality of microbiome data, variable screening in conjunction with penalized estimation may improve accuracy and algorithmic stability [14]. The sure independence screening (SIS) of Fan and Lv [14] retains features attaining the highest marginal correlation with the response, which may lead to poor performance when irrelevant features are more highly correlated with the response, marginally, than relevant ones. Since this is likely the case for microbiome data, we instead consider using the iterative sure independence screening procedure proposed by Fan and Lv [14] and implemented by Saldana and Feng [43] that avoids such a drawback by performing iterative feature recruitment and deletion based on a given penalty $p_{\lambda }$. Since features that are constant across all (or nearly all) samples are collinear with the model intercept, we recommend removing features with very low abundances such as those that are zero for most samples (e.g., Xiao et al. [67]).

### Post-selection inference and error rate control

Inference on which microbiome features have a population-level effect, conducted by testing the null hypothesis $H_{0j}: \beta _j = 0$ for the *j*th feature ($1\le j \le p)$, is challenging using penalized least squares estimation. For example, the asymptotic distribution of the LASSO may not be continuous and is difficult to characterize in high-dimensional settings [27]. Many approaches for error rate control post-variable selection using penalized regression make use of data splitting techniques [7, 12] but have low power for the small sample sizes common to microbiome studies. Due to these reasons, for inference we propose using the debiased, also known as desparsified, LASSO [56, 70] applied to the estimate $\hat{\varvec{\beta }}^l$ obtained using the LASSO penalty with the iterative SIS procedure. To make the computation tractable, we only apply the debiasing procedure to the features not screened out by the iterative SIS procedure and let $\hat{\varvec{b}}^l$ denote the resulting estimate. Under regularity assumptions and appropriate penalization, the debiased LASSO estimator has a limiting normal distribution [12].

For the *j*th feature, the standardized debiased iterative SIS-LASSO estimate ${\hat{b}}_j$ and its standard error are given by

5 $$\begin{aligned} {\hat{b}}_j = \sum _{l \in {\mathcal {L}}} {\hat{b}}_j^l\Pr (L_i = l) , \quad {\text {se}}({\widehat{b}}_j ) = \sqrt{\sum _{l \in {\mathcal {L}}} \left[ {\text {se}}({\hat{b}}_j^l)\Pr (L_i = l) \right] ^2}, \end{aligned}$$

respectively, where the standard error formula follows from the independence of the strata. To obtain an estimator of the standard error, we plug-in the estimate $\widehat{\text {se}}_j^l$ of ${\text {se}}({\hat{b}}_j^l)$ given by Dezeure et al. [11] under homoscedastic errors if the *j*th feature was not removed by screening in the *l*th confounder stratum. We compute a *p*-value for testing $H_{0j}: \beta _j = 0$ versus $H_{1j}: \beta _j \ne 0$ according to $p_j = 2[1 - \Phi ( |{\hat{b}}_j |/ \widehat{\text {se}}_j )]$ if feature *j* was not screened out in all confounder strata for $j= 1,\dots ,p$, where $\Phi (\cdot )$ denotes the standard normal cdf. To control the false discovery rate (FDR), we apply the Benjamini–Hochberg (BH) adjustment across all *p* features [4] to account for multiplicity in all features, including those that were removed from all strata.

## Simulation studies

Here, we evaluate our proposed standardization method using simulation studies. The simulation settings were designed to mimic microbiome studies seen in practice. To emulate species-level data, we consider $p = 2000$ microbiome features. To reflect data summarized at the genus level, we also consider $p = 50$. We consider sample sizes of $n = 50$ and $n=100$, and assume the confounder is a binary indicator that takes the value one for $i = 1, \dots , n/2$ and zero for $i = n/2 + 1, \dots , n$.

### Data-generating model for microbiome features

Conditional on the confounder $L_i = 0$, the count data for the *j*th microbiome feature were drawn independently from a negative binomial distribution with mean $\gamma _{0j}$ and dispersion $\phi _j$ parameterized such that ${{\,\mathrm{Var}\,}}( A_{ij} ) = \gamma _{0j} + \phi _j(\gamma _{0j})^2$. That is, when $L_i = 0$, the baseline mean for feature *j* is $\gamma _{0j}$. When the confounder is present ($L_i = 1$), the microbiome feature counts were drawn independently from a negative binomial distribution with mean $\gamma _{0j}\gamma _{1j}$ and dispersion $\phi _j$. Hence, $\gamma _{1j}$ represents the multiplicative change in the mean relative to when the confounder is absent. If $\gamma _{1j} \ne 1$, then feature *j* is affected by the confounder and otherwise $\gamma _{1j} = 1$. The first $30\%$ of features were set to be affected by the confounder (differentially abundant between condition $L_i = 0$ and condition $L_i = 1$). More specifically, we simulated parameters $\gamma _{0j}$ and $\gamma _{1j}$ from the following distributions for $j = 1,\dots , p$:

$$\begin{aligned}&\gamma _{0j} {\mathop {\sim }\limits ^{\text {ind}}}\left\{ \begin{array}{ll} \log {\mathcal {N}}\left( 1/2,~ 9/4 \right) &{}{\hbox { if}}\ \beta _j = 0 \\ \delta _{\{5\}} &{}{\hbox { if}}\ \beta _j \ne 0 \\ \end{array} \right. \\&\gamma _{1j} {\mathop {\sim }\limits ^{\text {ind}}}\left\{ \begin{array}{ll} \log {\mathcal {N}}\left( \pm 1/4,~ 9/4 \right) &{}{\hbox { if feature}}\; j \hbox { is affected by } L_i \\ \delta _{\{1\}} &{} {\text{ otherwise }} \\ \end{array} \right. \end{aligned}$$

where $\delta _{\{x\}}$ represents a point mass at *x*. Our rationale for setting the baseline mean to five for relevant features $(\beta _j \ne 0)$ was to ensure that they were sufficiently abundant for feature selection. We set the dispersions $\phi _j = 10^{-1}$ for all features $j = 1, \dots , p$ and simulated the microbiome count data ${\varvec{A}}_i$ with negative binomial distributions. In addition, we conducted a second set of simulations with $\phi _j = 10^{-6}$, which approximates a Poisson distribution.

### Data-generating model for response

Given the confounder and microbiome features ${\varvec{A}}_i$ simulated from the above subsection, we draw the responses independently from a normal distribution with mean $\mu _i(\tilde{\varvec{A}}_i, L_i)$ and variance $\sigma ^2$, where $\tilde{\varvec{A}_i}$ represents ${\varvec{A}}_i$ after centering and scaling (to mean zero and variance one within strata) and

6 $$\begin{aligned} \mu _i(\tilde{\varvec{A}}_i, L_i) = \left\{ \begin{array}{ll} \beta _0 + \sum _{j=1}^p {\tilde{A}}_{ij} \beta _j &{}{\hbox { if}}\ L_i = 0 \\ \beta _0 + \beta _\ell + \sum _{j=1}^p {\tilde{A}}_{ij} \delta \beta _j &{}{\hbox { if}}\ L_i = 1 \\ \end{array} \right. \end{aligned}$$

for $i = 1, \dots , n$. For more intuitive comparison of effect modification size, model (6) has an additive effect $\beta _\ell$ for the intercept and multiplicative effect $\delta$ for microbiome feature effects when $L_i = 1$ compared with $L_i =0$. In particular, $\beta _\ell$ represents the direct confounder effect and $\delta$ is an effect modification parameter. Our simulation considers the case when there is no effect modification ($\delta = 1$) as well as strong effect modification ($\delta = -0.9$) where the relevant microbiome effects are large within each level of the confounder but small overall in the population. The response variability was set to $\sigma ^2 = 1/16$ for all scenarios. A total of $s = 5$ features were set to be relevant, with the non-zero elements of ${\varvec{\beta }}$ set to $(3,-3,3,-3,3)$. Our motivation for setting $\left| \beta _j \right| = 3$ for all relevant *j* is to ensure the $\beta _{\min }$ property for model selection consistency is met within all strata for all simulation scenarios [7]. The choice of $s = 5$ yields sparsity such that $s < n_l / log(p)$ for most, but not all, simulation scenarios. Three scenarios covering differing proportions of the relevant features set to be confounded ($\beta _j \ne 0$ and $\gamma _{1j} \ne 1$) were considered: either all (100% confounded), the first three (60% confounded), or none (0% confounded).

To summarize our simulation settings, we have considered two dimensions of microbiome features: $p=2000$ and $p=50$; two sample sizes: $n=50$ and $n=100$; two distributions of microbiome count data: negative binomial and Poisson; inclusion of effect modifier: none or strong effect modifier; and three different proportions of confounded relevant features: 100%, 60%, and 0%. Hence, in total, we examined 48 different simulation settings. For each simulation setting, a total of 100 data sets were simulated.

### Screening, penalization, and comparison models

We denote our proposed approach of estimation conditional on each stratum followed by standardization as “Conditional Std”. We investigate the performance of variable section using the LASSO and SCAD penalties for $p_\lambda$ both with and without screening, as well as the proposed inference procedure using the debiased LASSO with iterative SIS described in "Post-selection inference and error rate control" section.

We compare our approach with existing penalized regression models applied to the pooled data set, as opposed to conditionally on each stratum. A total of six comparison models are constructed based on three inclusion strategies for the confounder effect $\beta _\ell$ of Eq. (6) and two possibilities for modeling effect modification. The confounder effect is either subject to screening and variable selection (“Select L”), forced to be included without penalization (“Require L”), or removed from the model entirely (“Ignore L”). We either model each microbiome feature effect as common across all confounder strata (corresponding to models with the aforementioned names) or allow for effect modification through stratum-specific microbiome feature effects denoted with the suffix “EffMod.” For each of the six models under comparison, we also investigate the performance of variable section using the LASSO and SCAD penalties for $p_\lambda$ both with and without screening, as well as the proposed inference procedure using the debiased LASSO with iterative SIS.

Table 1 presents the objective function for our proposed “Conditional Std“ approach and the other six models under comparison. For the proposed approach “Conditional Std,” screening is based on iterative SIS recommended defaults applied to each stratum, whereas for all other approaches it is applied to the entire data set to correspond with the assumed model, resulting in different maximum model sizes shown in Table 2. The variables considered in the iterative SIS procedure for each model detailed in Table 2 correspond to those penalized in the objective function in Table 1. For “Conditional Std” and models allowing effect modification (suffix “EffMod”), the population estimates are computed according to Eq. (4). These models center and scale each microbiome feature within each stratum, denoted by ${\tilde{A}}_{ij}$. For models that do not allow for effect modification, the microbiome features are centered and scaled to have mean zero and variance one across all observations, regardless of stratum, denoted by ${\dot{A}}_{ij}$.

### Results

Simulation performance was summarized across all 100 simulated data sets for each scenario, model, and variable selection method considered using the true positive rate (TPR) and false positive rate (FPR). Given the selected variables, TPR measures the proportion of relevant features detected, while FPR measures the proportion of irrelevant features declared to be relevant, and these are computed here at the population-level by

7 $$\begin{aligned} {\text {TPR}}= & {} \frac{\sum _{j=1}^pI({\hat{\beta }}_j \ne 0)I(\beta _j \ne 0)}{\sum _{j=1}^p I(\beta _j \ne 0)}, \end{aligned}$$

8 $$\begin{aligned} {\text {FPR}}= & {} \frac{\sum _{j=1}^pI({\hat{\beta }}_j \ne 0)I(\beta _j = 0)}{\sum _{j=1}^p I(\beta _j = 0)} \end{aligned}$$

for all methods except the debiased LASSO inference procedure where $I({\hat{\beta }}_j \ne 0)$ is replaced with the decision rule induced by the corresponding hypothesis test with FDR control at 0.05. An ideal method would take (TPR, FPR) values (1, 0). Additional file 1: Table S1 shows the average TPR and FPR across the 100 simulated data sets for the 12 simulation settings with Poisson distributed features and $n = 100$. The table lists the results for our proposed approach “Conditional Std” model and the other six models under comparison across different variable selection methods. Generally, the proposed “Conditional Std” model performed better than other models applied to the entire data set across different variable selection methods considered. When effect modification is present, the proposed approach has the highest mean TPR and lowest mean FPR for both the LASSO and SCAD penalties, both with and without screening, often achieving perfect rates on average. For the debiased LASSO applied after iterative SIS with the BH procedure and FDR control set to 0.05 (denoted by “iterSIS-dbLASSO-BH”), the proposed approach has the highest TPR and among the lowest FPR under strong effect modification across variable selection methods. This is not the case only when no effect modification is present, under high dimensionality ($p = 2000$), and not all relevant features are not confounded.

**Fig. 1:**
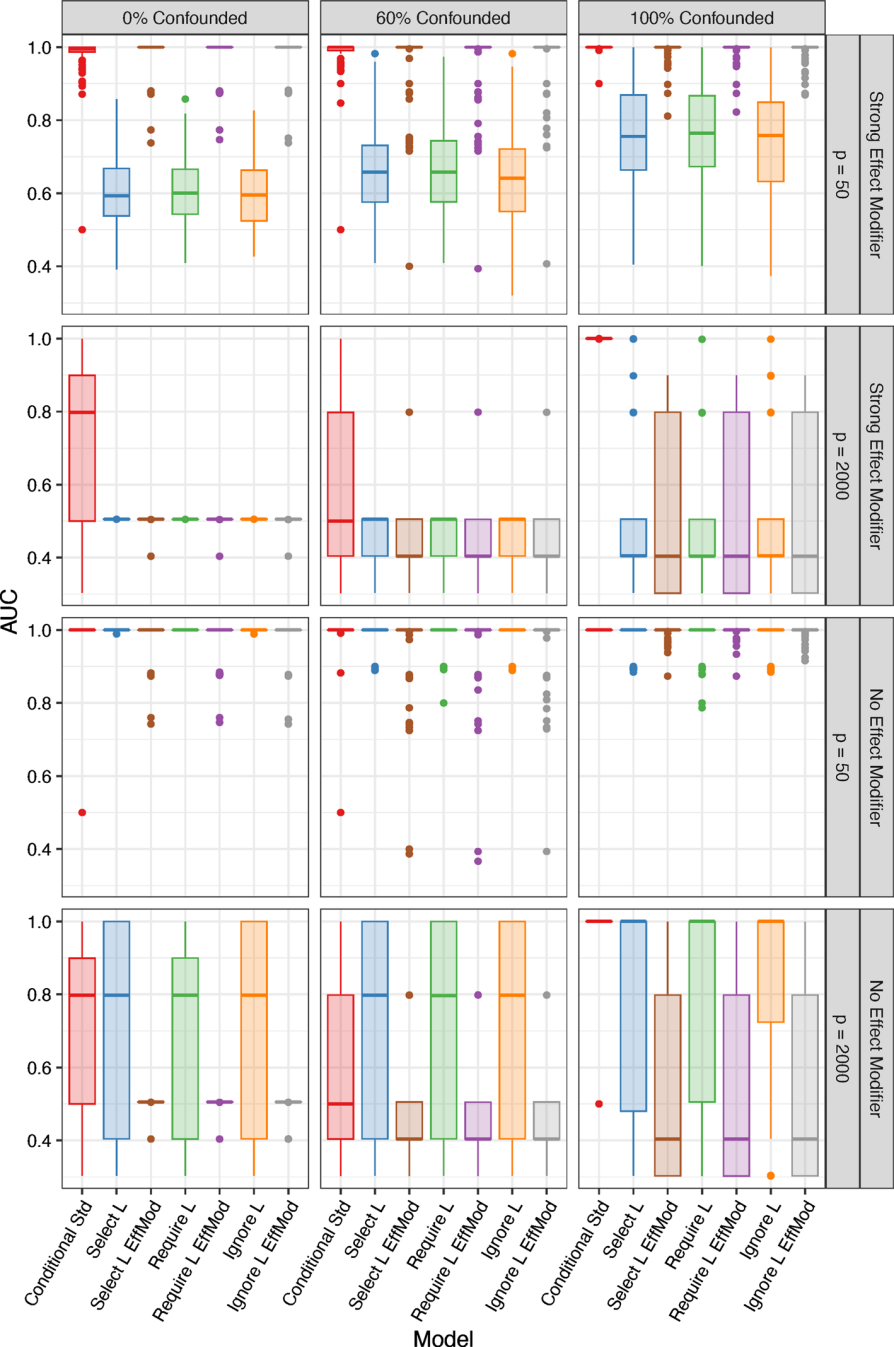
*Simulation results* Box plots of the area under the curve (AUC) from 100 simulation replications for $n = 100$ and Poisson features using *p*-values based on the debiased LASSO estimate following iterative sure independence screening

For post-selection inference based on the debiased LASSO following screening with iterative SIS, we evaluated the area under the receiver operating characteristic curve (AUC) using the *p*-values for testing $H_{0j}: \beta _j = 0$ as the classifiers. AUC aggregates classification performance of TPR versus FPR across different classification thresholds, taking the value 1 for perfect prediction, 0.5 for random guessing, and 0 for always wrong prediction. Box plots of the AUC across 100 data sets for each model are shown in Fig. 1 for 12 simulation settings with $n = 100$ and Poisson features (results for $n = 50$ and negative binomial features are presented in Additional file 1: Figs. S1–S3). The proposed approach has near perfect ranking under low dimensionality ($p = 50$) for all settings and under high dimensionality ($p=2000$) when all relevant features are impacted by the confounder. Similar to the results in Additional file 1: Table S1, the proposed approach performs best out of all models considered except when effect modification is not present and at least some relevant features are not confounded. Among the models that do not use a standardization approach, those that allow for effect modification (labeled with “EffMod”) perform better when there is an effect modifier in the data generation, whereas those that do not allow for effect modification perform better when there is no effect modifier in the data generation. For both cases, the proposed standardization approach is superior or competitive.

**Fig. 2:**
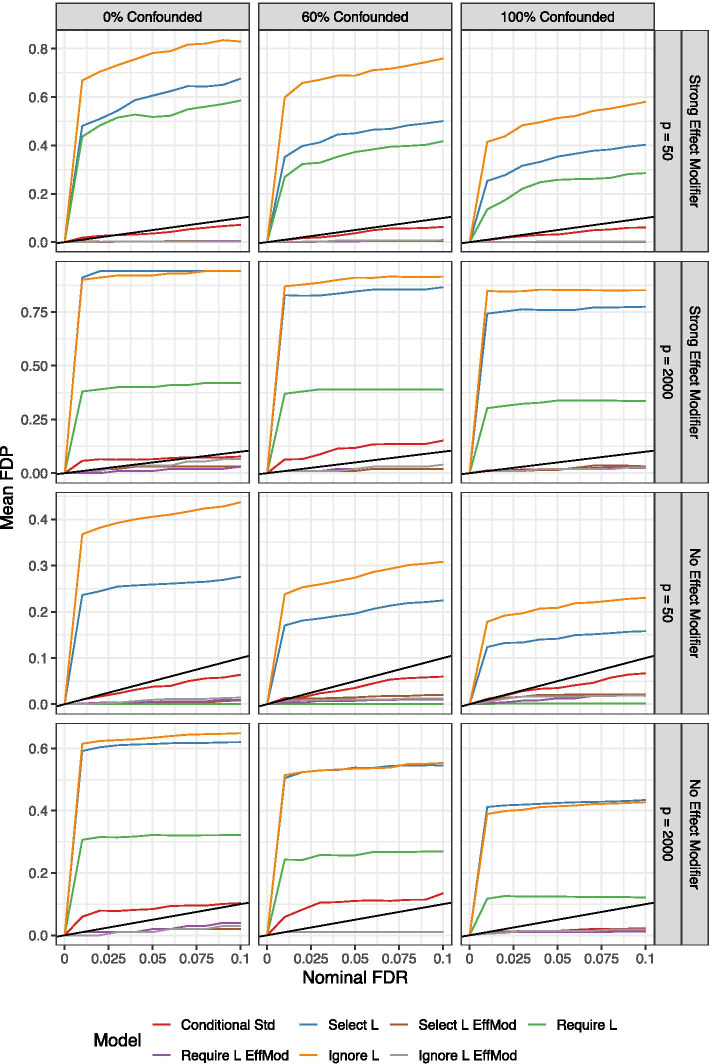
*Simulation results* Mean estimated false discovery proportion (FDP) for $n = 100$ and Poisson features at varying nominal false discovery rate (FDR) values using Benjamini–Hotchberg adjusted *p*-values based on the debiased LASSO estimate following iterative sure independence screening (iterative SIS). The $y = x$ line is shown in black; any values above this line indicate lack of FDR control

To evaluate false discovery rate (FDR) control for varying thresholds $\alpha = (0.01, 0.02, \dots , 0.10)$ commonly used in practice, we computed the false discovery proportion (FDP) at a given $\alpha$ value for debiased LASSO inference according to

9 $$\begin{aligned} {\text {FDP}}(\alpha ) = \frac{\sum _{j=1}^pI(q_{j}< \alpha )I(\beta _j = 0)}{\sum _{j=1}^p I(q_{j} < \alpha )}, \end{aligned}$$

where $q_j$ is the BH-adjusted *p*-value (or *q*-value) for feature *j*. A well performing model will have ${\text {FDP}}(\alpha ) \le \alpha$. For $n = 100$ and Poisson features, Fig. 2 shows that the proposed “Conditional Std” model appropriately controls FDR under low dimensionality ($p = 50$). For high dimensionality ($p = 2000$), the proposed approach does not control FDR when at least some relevant features are not confounded, though the observed mean FDP does not exceed the nominal level greatly when compared to other competing models applied to the pooled data. The FDR control for the other six models under comparison is either very conservative or highly liberal. Similar results were seen for $n = 50$ and negative binomial features, though lack of FDR control was more common for the $n = 50$ case (Additional file 1: Figs. S4–S6).

## Real data analysis

We conducted a microbiome study to investigate the effect of the rhizosphere microbiome of the cereal crop sorghum (*Sorghum bicolor*) on the phenotype 12-oxo phytodienoic acid (OPDA) production in the root. Sorghum root production of OPDA is of primary interest due to OPDA having both independent plant defense functions and being an important precursor to Jasmonic acid, which functions in plant immune responses that are induced by beneficial bacteria [57, 60]. The study analyzed here is part of an experiment described by Sheflin et al. [48]; we subset on $n = 34$ samples collected in September across high and low nitrogen fertilizer. Rhizosphere microbiome data were collected using 16S amplicon sequencing and clustered at 97% sequence identity. The resulting 5584 OTUs were rarefied to 20,000 reads per observation and low abundance OTUs (less than 4 non-zero observations out of 34) were excluded [67], leaving a total of 4244 OTUs.

Pairwise Spearman’s correlations for the feature counts are shown in Figure S7 in the Additional file 1 for the 150 largest marginal correlations (pooling samples over nitrogen fertilizer levels), which contrast to the small correlations within nitrogen stratum. Using our proposed procedure of testing the standardized feature effect using the debiased LASSO following iterative SIS applied to each nitrogen level, a total of four microbiome features with an effect on root ODPA production were identified while FDR was controlled at 0.05 with BH adjustment (Table 3). Nitrogen stratum-specific residuals did not indicate any violation of the assumptions of constant variance or normality (Figs. S8–S9 of the Additional file 1).

Each microbiome feature effect identified at the study population-level was only identified in one nitrogen condition, though abundance did not differ greatly between the two nitrogen strata (Table 3). Specifically, only one feature was estimated to be more abundant under low nitrogen, and this feature was classified as belonging to the *Rhodospirillaceae* family (nonsulfur photosynthetic bacteria), of which nearly all members have the capacity to fix molecular nitrogen [34]. Various strains of *Rhodospirillaceae* have shown potential to promote plant growth in the grass species *Brachiaria brizantha* [51]. Consequently, the increased levels of root OPDA content may have been the result of bacterial synthesis [15]. While less is known about the three additional significant features, the overall findings are in alignment with biological understanding of potential plant-microbe interactions.

**Table 1:** Models considered in simulation studies using penalized regression (with penalty $p_{\lambda }$) for a binary confounder $L_i \in \{0,1\}$

Model	Objective function
Conditional Std	$\sum _l\frac{1}{2n_l} \sum _i I(L_i=l) \left( y_i - \beta _0^l - \sum _j{\tilde{A}}_{ij} \beta _j^l\right) ^2 + \sum _{l,j} p_{\lambda _l}(\beta _j^l)$
Select L	$\frac{1}{2n} \sum _i \left( y_i - \beta _0 - L_i\beta _\ell - \sum _j {\dot{A}}_{ij} \beta _j\right) ^2 + \sum _j p_{\lambda }(\beta _j) + p_{\lambda }(\beta _\ell )$
Select L EffMod	$\frac{1}{2n} \sum _i\left( y_i - \beta _0 - L_i\beta _\ell - \sum _{l,j} I(L_i=l) {\tilde{A}}_{ij}\beta _j^l\right) ^2 + \sum _l\sum _{j} p_{\lambda }(\beta _j^l) + p_{\lambda }(\beta _\ell )$
Require L	$\frac{1}{2n} \sum _i \left( y_i - \beta _0 - L_i\beta _\ell - \sum _j {\dot{A}}_{ij} \beta _j\right) ^2 + \sum _j p_{\lambda }(\beta _j)$
Require L EffMod	$\frac{1}{2n} \sum _i\left( y_i - \beta _0 - L_i\beta _\ell - \sum _{l,j} I(L_i=l) {\tilde{A}}_{ij}\beta _j^l\right) ^2 + \sum _l\sum _j p_{\lambda }(\beta _j^l)$
Ignore L	$\frac{1}{2n} \sum _i \left( y_i - \beta _0 - \sum _j {\dot{A}}_{ij} \beta _j\right) ^2 + \sum _j p_{\lambda }(\beta _j)$
Ignore L EffMod	$\frac{1}{2n} \sum _i \left( y_i - \beta _0 - \sum _{l,j} I(L_i=l) {\tilde{A}}_{ij} \beta _j^l\right) ^2 + \sum _l\sum _j p_{\lambda }(\beta _j^l)$

${\tilde{A}}_{ij}$ denotes microbiome feature *j* centered and scaled within each stratum; ${\dot{A}}_{ij}$ denotes microbiome feature *j* centered and scaled across all observations, regardless of stratum

**Table 2:** Variable screening and selection for models considered in simulation studies for a binary confounder $L_i \in \{0,1\}$

Model	Variables screened	Maximum model size
Conditional Std	$\{\beta _1^l, \dots , \beta _p^l\}$ (independently $\forall l$)	$d_l = \lfloor n_l / \log (n_l)\rfloor$ $\forall l$
Select L	$\{\beta _1, \dots , \beta _p, L\}$	$d = \lfloor n / \log (n) \rfloor$
Select L EffMod	$\{\beta _1^{l=0}, \dots , \beta _p^{l=0}, \beta _1^{l=1}, \dots , \beta _p^{l=1}, L\}$	$d = \lfloor n / \log (n) \rfloor$
Require L	$\{\beta _1, \dots , \beta _p \}$ (given *L*)	$d = \lfloor n / \log (n) \rfloor$
Require L EffMod	$\{\beta _1^{l=0}, \dots , \beta _p^{l=0}, \beta _1^{l=1}, \dots , \beta _p^{l=1}\}$ (given *L*)	$d = \lfloor n / \log (n) \rfloor$
Ignore L	$\{\beta _1, \dots , \beta _p\}$	$d = \lfloor n / \log (n) \rfloor$
Ignore L EffMod	$\{\beta _1^{l=0}, \dots , \beta _p^{l=0}, \beta _1^{l=1}, \dots , \beta _p^{l=1}\}$	$d = \lfloor n / \log (n) \rfloor$

**Table 3:** Sorghum study analysis results: features with a significant effect on sorghum root ODPA production in the study population with FDR control at the 0.05 level using the Benjamini–Hochberg (BH) procedure on the debiased LASSO estimate following sure independence screening (iterative SIS)

	Standardized		Conditional: high N			Conditional: low N		
Feature	Estimate	*q*-value	Estimate	*q*-value	Mean (SD)	Estimate	*q*-value	Mean (SD)
Order	3.18	$<0.001$	6.36	$<0.001$	56.8 (10.6)	0.00	1.000	52.5 (10.2)
*Rhodocyclales*								
Family	4.68	$<0.001$	0.00	1.000	13.4 (4.0)	9.37	$<0.001$	5.1 (2.2)
*Rhodospirillaceae*								
Genus	3.72	$<0.001$	7.45	$<0.001$	6.9 (6.5)	0.00	1.000	1.6 (1.5)
*Massilia*								
Unnamed order	1.53	0.001	3.06	0.001	6.7 (3.5)	0.00	1.000	10.9 (5.8)
Sva0725								

## Discussion

We have proposed and evaluated methodology for causal inference for individual features in high-dimensional microbiome data using standardization. These techniques are typically employed in epidemiology and use the potential-outcomes framework, in contrast to graphical models, which are a more common approach for high-dimensional causal inference but usually require Gaussian assumptions for inference that are often violated by microbiome data [38]. Instead, our approach conditions on the confounder and shows favorable results for Poisson and negative binomial microbiome features. Compared to estimation methods applied to the entire data set, the proposed standardization approach typically demonstrated superior recovery of relevant microbiome effects accross multiple variable screening and selection procedures.

Association and causation are not equivalent even for a one-dimensional treatment or exposure, and the challenges of causal analysis are exacerbated for high-dimensional exposures. Caution must be taken in interpreting causal effects when the assumptions needed for causal inference, such as no unmeasured confounding or consistency, cannot be verified. Consequently, any microbiome features identified should be either validated in experimental studies if possible, or more closely scrutinized according to guidelines for evidence of causation. However, even if conditions for causal inference do not hold, our method may provide better recovery of associational microbiome effects as compared to models applied to the pooled data, when there are features impacted by the confounder.

Some have advocated that microbiome data must be treated as compositional [17]. Due to the sum to library size constraint, which is not removed by rarefying but rather made constant across all samples, microbiome data technically lie in a simplex space [1]. One goal of our funded project is to identify microbial features that can be intervened upon to produce a favorable outcome. Hence we analyze count data, not compositional data where it is impossible to alter a feature without changing at least one other so as to retain the same total sum across features. When microbiome features are high dimensional, and in particular there is no dominating feature, the impact of this issue may be minimal. Moreover, microbiome data often exhibit many zeros and the popular centered log-ratio approach for compositional data applies log transformation after adding an arbitrary pseudocount, the choice of which may impact the analysis [10]. In cases when compositional analysis is preferred, such as when taxa are summarized at the level of genus or higher typically leading to $p < n$ with a lower prevalence of zeros, our strategy of standardization could be altered in a straightforward way by replacing penalized least squares with a regularized method for compositional covariates [31, 49].

Depending on the underlying biology, the taxonomic structure or phylogeny may be important in the relationship between the microbiome and outcome. If so, higher power may be achieved by using a different penalty that leverages such information. The group LASSO selects groups of features [68] and modifications have been developed for microbiome applications incorporating multiple levels of taxonomic hierarchy [16]. Other options include a phylogeny-based penalty that penalizes coefficients along a supplied phylogenetic tree [66] or a kernel-based penalty incorporating a desired ecological distance [39]. To increase power and address the challenge of FDR control, the hierarchical taxonomic structure could be utilized in a multi-stage FDR controlling approach [23]. Applications of these methods require the taxa assignments and phylogenetic tree, which may be incompletely elucidated for novel microbial species, or measured with error [18, 32].

While simulation studies showed our proposed approach had higher power and better control of FDR at the nominal level compared to other approaches for most scenarios considered, use of the BH procedure with the debiased LASSO and the iterative SIS procedure failed to control FDR for some cases under high dimensionality. Recently, Javanmard and Javadi [25] showed that the BH procedure may fail to control FDR using the debiased LASSO due to correlation between estimates, but we found little indication of highly correlated estimates in our simulation studies. Correspondingly, applying the Benjamini–Yekutieli adjustment [5] did not result in better FDR control. Instead, it appears our sample sizes were too small to achieve a high enough probability of the sure screening property, leading to relevant features being screened out by the iterative SIS procedure. While additional methodological advancement is needed for valid inference following both variable screening and selection when sample sizes are small, our method performed competitively in recovering relevant features.

## Conclusion

We have addressed the problem of selecting microbiome features relevant to an outcome of interest under confounding by a categorical variable. Our results indicate that standardization enables more accurate identification of individual microbiome features with an effect on the outcome of interest compared to other variable selection and estimation procedures.

## Supplementary information

**Additional file 1**. Additional simulation results and data analysis.

**Additional file 2: R Code**. R and R markdown code for all simulation studies and data analysis.

## Data Availability

R and R markdown code for simulation studies and data analysis are provided in Additional file 2: R Code. Sequence data can be found in the NCBI SRA submission library under the following accession numbers: sequencing project IDs #1095844, #1095845, #1095846; SRA identifier #SRP165130.

## References

[1] Aitchison J (1982). The statistical analysis of compositional data. J R Stat Soc Ser B (Methodol).

[2] Baksi KD, Kuntal BK, Mande SS (2018). TIME: a web application for obtaining insights into microbial ecology using longitudinal microbiome data. Front Microbiol.

[3] Banerjee S, Schlaeppi K, van der Heijden MGA (2018). Keystone taxa as drivers of microbiome structure and functioning. Nat Rev Microbiol.

[4] Benjamini Y, Hochberg Y (1995). Controlling the false discovery rate: a practical and powerful approach to multiple testing. J R Stat Soc Ser B (Methodol).

[5] Benjamini Y, Yekutieli D (2001). The control of the false discovery rate in multiple testing under dependency. Ann Stat.

[6] Boers SA, Jansen R, Hays JP (2016). Suddenly everyone is a microbiota specialist. Clin Microbiol Infect.

[7] Bühlmann P, Kalisch M, Meier L (2014). High-dimensional statistics with a view toward applications in biology. Annu Rev Stat Appl.

[8] Callahan BJ, McMurdie PJ, Holmes SP (2017). Exact sequence variants should replace operational taxonomic units in marker-gene data analysis. ISME J.

[9] Camacho-Ortiz A, Gutiérrez-Delgado EM, Garcia-Mazcorro JF, Mendoza-Olazarán S, Martínez-Meléndez A, Palau-Davila L, Baines SD, Maldonado-Garza H, Garza-González E (2017). Randomized clinical trial to evaluate the effect of fecal microbiota transplant for initial Clostridium difficile infection in intestinal microbiome. PLoS ONE.

[10] Costea PI, Zeller G, Sunagawa S, Bork P (2014). A fair comparison. Nat Methods.

[11] Dezeure R, Bühlmann P, Zhang C-H (2017). High-dimensional simultaneous inference with the bootstrap. TEST.

[12] Dezeure R, Bühlmann P, Meier L, Meinshausen N (2015). High-dimensional inference: confidence intervals, p-values and R-software hdi. Stat Sci.

[13] Fan J, Li R (2001). Variable selection via nonconcave penalized likelihood and its oracle properties. J Am Stat Assoc.

[14] Fan J, Lv J (2008). Sure independence screening for ultrahigh dimensional feature space. J R Stat Soc Ser (Stat Methodol).

[15] Forchetti G, Masciarelli O, Alemano S, Alvarez D, Abdala G (2007). Endophytic bacteria in sunflower (Helianthus annuus l.): isolation, characterization, and production of jasmonates and abscisic acid in culture medium. Appl Microbiol Biotechnol.

[16] Garcia TP, Müller S, Carroll RJ, Walzem RL (2014). Identification of important regressor groups, subgroups and individuals via regularization methods: application to gut microbiome data. Bioinformatics.

[17] Gloor GB, Macklaim JM, Pawlowsky-Glahn V, Egozcue JJ (2017). Microbiome datasets are compositional: And this is not optional. Front Microbiol.

[18] Golob JL, Margolis E, Hoffman NG, Fredricks DN (2017). Evaluating the accuracy of amplicon-based microbiome computational pipelines on simulated human gut microbial communities. BMC Bioinform.

[19] Granger CWJ (1969). Investigating causal relations by econometric models and cross-spectral methods. Econometrica.

[20] Hernán MA, Robins JM (2019). Causal inference.

[21] Hernán MA, Brumback B, Robins JM (2001). Marginal structural models to estimate the joint causal effect of nonrandomized treatments. J Am Stat Assoc.

[22] Holland PW (1988). Causal inference, path analysis, and recursive structural equations models. Sociol Methodol.

[23] Hu J, Koh H, He L, Liu M, Blaser MJ, Li H (2018). A two-stage microbial association mapping framework with advanced FDR control. Microbiome.

[24] Imai K, Van Dyk DA (2004). Causal inference with general treatment regimes: generalizing the propensity score. J Am Stat Assoc.

[25] Javanmard A, Javadi H (2019). False discovery rate control via debiased lasso. Electron J Stat.

[26] Keiding N, Clayton D (2014). Standardization and control for confounding in observational studies: a historical perspective. Stat Sci.

[27] Knight K, Fu W (2000). Asymptotics for lasso-type estimators. Ann Stat.

[28] Knight R, Vrbanac A, Taylor BC, Aksenov A, Callewaert C, Debelius J, Gonzalez A, Kosciolek T, McCall L-I, McDonald D (2018). Best practices for analysing microbiomes. Nat Rev Microbiol.

[29] Lederberg J, Mccray AT (2001). Ome SweetOmics: a genealogical treasury of words. Scientist.

[30] Leng C, Lin Y, Wahba G (2006). A note on the lasso and related procedures in model selection. Stat Sin.

[31] Lin W, Shi P, Feng R, Li H (2014). Variable selection in regression with compositional covariates. Biometrika.

[32] Lindgreen S, Adair KL, Gardner PP (2016). An evaluation of the accuracy and speed of metagenome analysis tools. Sci Rep.

[33] Liu L, Li Y, Li S, Hu N, He Y, Pong R, Lin D, Lu L, Law M. Comparison of next-generation sequencing systems. J Biomed Biotechnol. 2012. 10.1155/2012/251364 PMC3398667 22829749

[34] Madigan M, Cox SS, Stegeman RA (1984). Nitrogen fixation and nitrogenase activities in members of the family rhodospirillaceae. J Bacteriol.

[35] McMurdie PJ, Holmes S (2014). Waste not, want not: why rarefying microbiome data is inadmissible. PLoS Comput Biol.

[36] Nandy P, Maathuis MH, Richardson TS (2017). Estimating the effect of joint interventions from observational data in sparse high-dimensional settings. Ann Stat.

[37] Neyman J (1923). On the application of probability theory to agricultural experiments. Essay on principles. Section 9. Stat Sci.

[38] Pearl J (2009). Causality models: reasoning and inference.

[39] Randolph TW, Zhao S, Copeland W, Hullar M, Shojaie A (2018). Kernel-penalized regression for analysis of microbiome data. Ann Appl Stat.

[40] Riesenfeld CS, Schloss PD, Handelsman J (2004). Metagenomics: genomic analysis of microbial communities. Annu Rev Genet.

[41] Rogers GB, Stressmann FA, Koller G, Daniels T, Carroll MP, Bruce KD (2008). Assessing the diagnostic importance of nonviable bacterial cells in respiratory infections. Diagn Microbiol Infect Dis.

[42] Rubin DB (1974). Estimating causal effects of treatments in randomized and nonrandomized studies. J Educ Psychol.

[43] Saldana D, Feng Y (2018). SIS: an R package for sure independence screening in ultrahigh-dimensional statistical models. J Stat Softw.

[44] Schloss PD, Westcott SL (2011). Assessing and improving methods used in operational taxonomic unit-based approaches for 16S rRNA gene sequence analysis. Appl Environ Microbiol.

[45] Schneeweiss S, Rassen JA, Glynn RJ, Avorn J, Mogun H, Brookhart MA (2009). High-dimensional propensity score adjustment in studies of treatment effects using health care claims data. Epidemiology (Cambridge, Mass).

[46] Schwarz G (1978). Estimating the dimension of a model. Ann Stat.

[47] Sharpton TJ (2014). An introduction to the analysis of shotgun metagenomic data. Front Plant Sci.

[48] Sheflin AM, Chiniquy D, Yuan C, Goren E, Kumar I, Braud M, Brutnell T, Eveland AL, Tringe S, Liu P, Kresovich S, Marsh EL, Schachtman DP, Prenni JE (2019). Metabolomics of sorghum roots during nitrogen stress reveals compromised metabolic capacity for salicylic acid biosynthesis. Plant Direct.

[49] Shi P, Zhang A, Li H (2016). Regression analysis for microbiome compositional data. Ann Appl Stat.

[50] Siddique AA, Schnitzer ME, Bahamyirou A, Wang G, Holtz TH, Migliori GB, Sotgiu G, Gandhi NR, Vargas MH, Menzies D (2018). Causal inference with multiple concurrent medications: a comparison of methods and an application in multidrug-resistant tuberculosis. Stat Methods Med Res.

[51] Silva MCP, Figueiredo AF, Andreote FD, Cardoso EJBN (2013). Plant growth promoting bacteria in brachiaria brizantha. World J Microbiol Biotechnol.

[52] Sohn MB, Li H (2019). Compositional mediation analysis for microbiome studies. Ann Appl Stat.

[53] Stewart EJ (2012). Growing unculturable bacteria. J Bacteriol.

[54] Taubman SL, Robins JM, Mittleman MA, Hernán MA (2009). Intervening on risk factors for coronary heart disease: an application of the parametric g-formula. Int J Epidemiol.

[55] Tibshirani R (1996). Regression shrinkage and selection via the lasso. J R Stat Soc Ser B (Methodol).

[56] van de Geer S, Bühlmann P, Ritov Y, Dezeure R (2014). On asymptotically optimal confidence regions and tests for high-dimensional models. Ann Stat.

[57] Van der Ent S, Van Wees SC, Pieterse CM (2009). Jasmonate signaling in plant interactions with resistance-inducing beneficial microbes. Phytochemistry.

[58] Wang C, Hu J, Blaser MJ, Li H (2019). Estimating and testing the microbial causal mediation effect with high-dimensional and compositional microbiome data. Bioinformatics.

[59] Wang H, Li R, Tsai C-L (2007). Tuning parameter selectors for the smoothly clipped absolute deviation method. Biometrika.

[60] Wasternack C (2014). Action of jasmonates in plant stress responses and development-applied aspects. Biotechnol Adv.

[61] Weiss S, Xu ZZ, Peddada S, Amir A, Bittinger K, Gonzalez A, Lozupone C, Zaneveld JR, Vázquez-Baeza Y, Birmingham A, Hyde ER, Knight R (2017). Normalization and microbial differential abundance strategies depend upon data characteristics. Microbiome.

[62] Westcott SL, Schloss PD (2015). De novo clustering methods outperform reference-based methods for assigning 16S rRNA gene sequences to operational taxonomic units. PeerJ.

[63] Westreich D, Cole SR (2010). Invited commentary: positivity in practice. Am J Epidemiol.

[64] Wilson A, Zigler CM, Patel CJ, Dominici F (2018). Model-averaged confounder adjustment for estimating multivariate exposure effects with linear regression. Biometrics.

[65] Xia Y, Sun J (2017). Hypothesis testing and statistical analysis of microbiome. Genes Dis.

[66] Xian J, Chen L, Yu Y, Zhang X, Chen J (2018). A phylogeny-regularized sparse regression model for predictive modeling of microbial community data. Front Microbiol.

[67] Xiao J, Chen L, Johnson S, Yu Y, Zhang X, Chen J (2018). Predictive modeling of microbiome data using a phylogeny-regularized generalized linear mixed model. Front Microbiol.

[68] Yuan M, Lin Y (2006). Model selection and estimation in regression with grouped variables. J R Stat Soc Ser B (Stat Methodol).

[69] Zhang C-H (2010). Nearly unbiased variable selection under minimax concave penalty. Ann Stat.

[70] Zhang C-H, Zhang SS (2014). Confidence intervals for low dimensional parameters in high dimensional linear models. J R Stat Soc Ser B (Stat Methodol).

[71] Zhang J, Wei Z, Chen J (2018). A distance-based approach for testing the mediation effect of the human microbiome. Bioinformatics.

