# Supplementary material for: Feature selection and causal analysis for microbiome studies in the presence of confounding using standardization
Source: BMC Bioinformatics. 2021 Jul 6;22:362. doi: 10.1186/s12859-021-04232-2 (PMC8261956; doi:10.1186/s12859-021-04232-2)
Supplement: Supplementary file 1 — Additional file 1. Additional simulation results and data analysis. [file 12859_2021_4232_MOESM1_ESM.pdf]

Supplementary Material:
Feature selection and causal analysis for microbiome studies in the presence of confounding using standardization

Emily Goren

May 2, 2021

Contents

S1 Additional simulation results **2**

S2 Real data analysis: correlation structure and assumption checks **9**

List of Tables

S1 Simulation results: TPR and FPR, $n = 100$, Poisson features 2

List of Figures

S1 Simulation results: AUC, $n = 50$, Poisson features 3
S2 Simulation results: AUC, $n = 50$, negative binomial features 4
S3 Simulation results: AUC, $n = 100$, negative binomial features 5
S4 Simulation results: FDP, $n = 50$, Poisson features 6
S5 Simulation results: FDP, $n = 50$, negative binomial features 7
S6 Simulation results: FDP, $n = 100$, negative binomial features 8
S7 Sorghum data: pairwise OTU correlations 9
S8 Sorghum data: conditional residuals vs. predicted 9
S9 Sorghum data: conditional residual Q-Q plot 10

S1 Additional simulation results

Table S1: Simulation results: true positive rate (TPR) and false positive rate (FPR) for identification of population coefficients for $n = 100$ and Poisson features, reported as means over 100 simulation replications. For the debiased LASSO with iterative SIS and BH procedure (labeled "iterSIS-dbLASSO-BH"), FDR control was at the 0.05 level.

		Variable Selection Method									
		iterSIS-dbLASSO-BH		iterSIS-LASSO		iterSIS-SCAD		LASSO		SCAD	
Simulation Scenario	Model	TPR	FPR	TPR	FPR	TPR	FPR	TPR	FPR	TPR	FPR
Strong Effect Modifier,	Conditional Std	**1.00**	0.02	**1.00**	**0.05**	**1.00**	**0.02**	**1.00**	**0.02**	**1.00**	**0.00**
$p = 50$, 0% confounded	Select L	0.04	0.04	0.96	0.96	0.87	0.80	0.96	0.96	0.87	0.80
	Select L EffMod	0.96	**0.00**	0.96	0.08	0.97	0.05	**1.00**	0.03	**1.00**	**0.00**
	Require L	0.03	0.03	0.95	0.95	0.86	0.81	0.95	0.95	0.86	0.81
	Require L EffMod	0.96	**0.00**	0.96	0.08	0.97	0.05	**1.00**	**0.02**	**1.00**	**0.00**
	Ignore L	0.09	0.09	0.96	0.95	0.86	0.80	0.96	0.95	0.87	0.80
	Ignore L EffMod	0.97	**0.00**	0.97	0.08	0.97	0.04	**1.00**	**0.02**	**1.00**	**0.00**
Strong Effect Modifier,	Conditional Std	**1.00**	0.01	**1.00**	**0.04**	**0.99**	**0.02**	**1.00**	**0.02**	**1.00**	**0.00**
$p = 50$, 60% confounded	Select L	0.18	0.05	0.97	0.95	0.81	0.79	0.97	0.95	0.81	0.79
	Select L EffMod	0.90	**0.00**	0.95	0.09	0.96	0.05	**1.00**	0.03	**1.00**	**0.00**
	Require L	0.13	0.03	0.95	0.94	0.81	0.79	0.95	0.94	0.81	0.79
	Require L EffMod	0.89	**0.00**	0.96	0.08	0.96	0.04	**1.00**	**0.02**	**1.00**	**0.00**
	Ignore L	0.29	0.10	0.95	0.94	0.82	0.79	0.95	0.94	0.82	0.79
	Ignore L EffMod	0.90	**0.00**	0.96	0.09	0.97	0.05	**1.00**	**0.02**	**1.00**	**0.00**
Strong Effect Modifier,	Conditional Std	**1.00**	0.01	**1.00**	**0.02**	**1.00**	**0.00**	**1.00**	**0.02**	**1.00**	**0.00**
$p = 50$, 100% confounded	Select L	0.32	0.04	0.94	0.91	0.81	0.71	0.95	0.91	0.81	0.71
	Select L EffMod	0.91	**0.00**	0.99	0.06	**1.00**	0.03	**1.00**	**0.02**	**1.00**	**0.00**
	Require L	0.24	0.02	0.93	0.89	0.81	0.72	0.93	0.89	0.81	0.72
	Require L EffMod	0.92	**0.00**	0.99	0.06	**1.00**	0.02	**1.00**	**0.02**	**1.00**	**0.00**
	Ignore L	0.44	0.07	0.92	0.89	0.81	0.71	0.92	0.89	0.81	0.71
	Ignore L EffMod	0.92	**0.00**	0.99	0.06	**1.00**	0.02	**1.00**	**0.02**	**1.00**	**0.00**
Strong Effect Modifier,	Conditional Std	**0.55**	**0.00**	**0.61**	**0.01**	**0.63**	**0.01**	**1.00**	**0.01**	**1.00**	**0.00**
$p = 2000$, 0% confounded	Select L	0.00	**0.00**	0.00	**0.01**	0.00	**0.01**	0.05	0.05	0.02	0.02
	Select L EffMod	0.01	**0.00**	0.01	**0.01**	0.01	**0.01**	**1.00**	**0.01**	**1.00**	**0.00**
	Require L	0.00	**0.00**	0.00	**0.01**	0.00	**0.01**	0.05	0.05	0.02	0.02
	Require L EffMod	0.01	**0.00**	0.01	**0.01**	0.00	**0.01**	**1.00**	**0.01**	**1.00**	**0.00**
	Ignore L	0.00	0.01	0.00	**0.01**	0.00	**0.01**	0.05	0.05	0.02	0.02
	Ignore L EffMod	0.00	**0.00**	0.00	**0.01**	0.01	**0.01**	**1.00**	**0.01**	**1.00**	**0.00**
Strong Effect Modifier,	Conditional Std	**0.48**	**0.00**	**0.52**	**0.01**	**0.54**	**0.01**	**0.99**	**0.01**	**1.00**	**0.00**
$p = 2000$, 60% confounded	Select L	0.06	**0.00**	0.09	**0.01**	0.09	**0.01**	0.09	0.05	0.07	0.02
	Select L EffMod	0.11	**0.00**	0.15	**0.01**	0.17	**0.01**	**0.99**	**0.01**	**1.00**	**0.00**
	Require L	0.01	**0.00**	0.09	**0.01**	0.09	**0.01**	0.10	0.04	0.06	0.02
	Require L EffMod	0.13	**0.00**	0.15	**0.01**	0.17	**0.01**	**0.99**	**0.01**	**1.00**	**0.00**
	Ignore L	0.07	0.01	0.09	**0.01**	0.10	**0.01**	0.09	0.05	0.07	0.02
	Ignore L EffMod	0.12	**0.00**	0.16	**0.01**	0.17	**0.01**	**0.99**	**0.01**	**1.00**	**0.00**
Strong Effect Modifier,	Conditional Std	**1.00**	**0.00**	**1.00**	**0.00**	**1.00**	**0.00**	**0.98**	**0.01**	**1.00**	**0.00**
$p = 2000$, 100% confounded	Select L	0.16	**0.00**	0.20	0.01	0.24	0.01	0.17	0.04	0.11	0.02
	Select L EffMod	0.30	**0.00**	0.36	0.01	0.38	0.01	**0.98**	**0.01**	**1.00**	**0.00**
	Require L	0.03	**0.00**	0.22	0.01	0.24	0.01	0.17	0.04	0.11	0.02
	Require L EffMod	0.32	**0.00**	0.36	0.01	0.37	0.01	**0.98**	**0.01**	**1.00**	**0.00**
	Ignore L	0.19	0.01	0.22	0.01	0.25	0.01	0.17	0.04	0.11	0.02
	Ignore L EffMod	0.31	**0.00**	0.37	0.01	0.39	0.01	**0.98**	**0.01**	**1.00**	**0.00**
No Effect Modifier,	Conditional Std	**1.00**	0.02	**1.00**	0.05	**1.00**	**0.02**	**1.00**	0.02	**1.00**	**0.00**
$p = 50$, 0% confounded	Select L	**1.00**	0.06	**1.00**	0.14	**1.00**	0.05	**1.00**	0.14	**1.00**	0.05
	Select L EffMod	0.97	**0.00**	0.97	0.08	0.97	0.04	**1.00**	0.03	**1.00**	**0.00**
	Require L	**1.00**	**0.00**	**1.00**	**0.00**	**1.00**	0.04	**1.00**	**0.00**	**1.00**	0.04
	Require L EffMod	0.97	**0.00**	0.97	0.08	0.97	0.04	**1.00**	0.02	**1.00**	**0.00**
	Ignore L	**1.00**	0.11	**1.00**	0.14	**1.00**	0.05	**1.00**	0.14	**1.00**	0.05
	Ignore L EffMod	0.97	**0.00**	0.97	0.09	0.97	0.04	**1.00**	0.02	**1.00**	**0.00**
No Effect Modifier,	Conditional Std	**1.00**	0.01	**1.00**	0.04	0.99	**0.02**	**1.00**	**0.02**	**1.00**	**0.00**
$p = 50$, 60% confounded	Select L	0.99	0.05	0.99	0.13	**1.00**	0.03	0.99	0.13	**1.00**	0.03
	Select L EffMod	0.77	**0.00**	0.96	0.09	0.96	0.05	**1.00**	**0.02**	**1.00**	**0.00**
	Require L	0.97	**0.00**	0.97	**0.03**	**1.00**	0.03	0.97	0.03	**1.00**	0.03
	Require L EffMod	0.77	**0.00**	0.96	0.08	0.96	0.05	**1.00**	**0.02**	**1.00**	**0.00**
	Ignore L	0.99	0.07	0.99	0.12	**1.00**	0.03	0.99	0.12	**1.00**	0.03
	Ignore L EffMod	0.76	**0.00**	0.96	0.09	0.96	0.05	**1.00**	**0.02**	**1.00**	**0.00**
No Effect Modifier,	Conditional Std	**1.00**	0.02	**1.00**	**0.02**	**1.00**	**0.00**	**1.00**	**0.02**	**1.00**	**0.00**
$p = 50$, 100% confounded	Select L	0.98	0.04	0.98	0.13	0.99	0.03	0.98	0.14	0.99	0.03
	Select L EffMod	0.70	0.01	**1.00**	0.07	**1.00**	0.03	**1.00**	0.03	0.99	**0.00**
	Require L	0.95	**0.00**	0.96	0.05	0.99	0.02	0.96	0.05	0.99	0.02
	Require L EffMod	0.70	**0.00**	**1.00**	0.06	**1.00**	0.02	**1.00**	**0.02**	**1.00**	**0.00**
	Ignore L	0.98	0.05	0.98	0.13	0.99	0.03	0.98	0.13	0.99	0.03
	Ignore L EffMod	0.69	**0.00**	**1.00**	0.06	**1.00**	0.02	**1.00**	**0.02**	**1.00**	**0.00**
No Effect Modifier,	Conditional Std	0.55	**0.00**	**0.61**	**0.01**	0.63	**0.01**	**1.00**	0.01	**1.00**	**0.00**
$p = 2000$, 0% confounded	Select L	0.60	**0.00**	**0.61**	**0.01**	0.64	**0.01**	**1.00**	0.01	**1.00**	**0.00**
	Select L EffMod	0.01	**0.00**	0.01	**0.01**	0.01	**0.01**	**1.00**	0.01	**1.00**	**0.00**
	Require L	0.40	**0.00**	0.57	**0.01**	0.60	**0.01**	**1.00**	**0.00**	**1.00**	**0.00**
	Require L EffMod	0.01	**0.00**	0.01	**0.01**	0.01	**0.01**	**1.00**	0.01	**1.00**	**0.00**
	Ignore L	**0.61**	**0.00**	**0.61**	**0.01**	**0.65**	**0.01**	**1.00**	0.01	**1.00**	**0.00**
	Ignore L EffMod	0.01	**0.00**	0.01	**0.01**	0.01	**0.01**	**1.00**	0.01	**1.00**	**0.00**
No Effect Modifier,	Conditional Std	0.48	**0.00**	0.53	**0.01**	0.54	**0.01**	**0.99**	0.01	**1.00**	**0.00**
$p = 2000$, 60% confounded	Select L	0.54	**0.00**	0.56	**0.01**	0.62	**0.01**	0.96	0.01	0.97	**0.00**
	Select L EffMod	0.11	**0.00**	0.19	**0.01**	0.19	**0.01**	**0.99**	0.01	**1.00**	**0.00**
	Require L	0.42	**0.00**	0.55	**0.01**	0.59	**0.01**	0.92	**0.00**	0.97	**0.00**
	Require L EffMod	0.12	**0.00**	0.19	**0.01**	0.19	**0.01**	**0.99**	0.01	**1.00**	**0.00**
	Ignore L	**0.56**	**0.00**	**0.59**	**0.01**	**0.65**	**0.01**	0.96	0.01	0.97	**0.00**
	Ignore L EffMod	0.12	**0.00**	0.20	**0.01**	0.20	**0.01**	**0.99**	0.01	**1.00**	**0.00**
No Effect Modifier,	Conditional Std	**1.00**	**0.00**	**1.00**	**0.00**	**1.00**	**0.00**	**0.99**	**0.01**	**1.00**	**0.00**
$p = 2000$, 100% confounded	Select L	0.75	**0.00**	0.76	0.01	0.82	**0.00**	0.91	**0.01**	0.92	**0.00**
	Select L EffMod	0.23	**0.00**	0.44	0.01	0.45	0.01	**0.99**	**0.01**	0.99	**0.00**
	Require L	0.65	**0.00**	0.73	**0.00**	0.78	**0.00**	0.83	**0.01**	0.94	**0.00**
	Require L EffMod	0.24	**0.00**	0.44	0.01	0.46	0.01	0.98	**0.01**	**1.00**	**0.00**
	Ignore L	0.78	**0.00**	0.78	0.01	0.88	**0.00**	0.91	**0.01**	0.92	**0.00**
	Ignore L EffMod	0.23	**0.00**	0.44	0.01	0.45	0.01	0.98	**0.01**	**1.00**	**0.00**

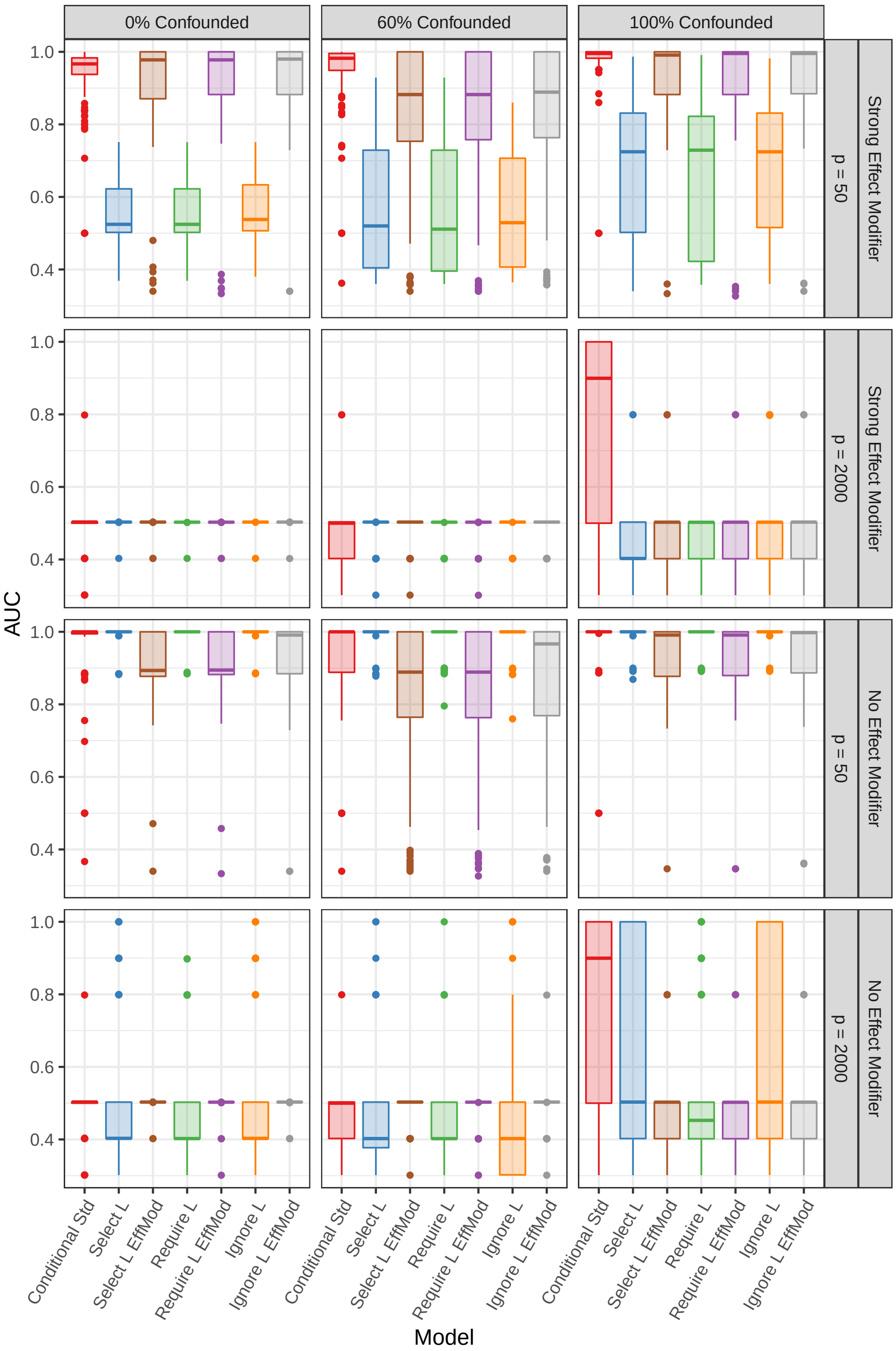

Figure S1: Simulation results: box plots of the area under the curve (AUC) from 100 simulation replications for $n = 50$ and Poisson features using p-values based on the debiased LASSO estimate following iterative sure independence screening (iterative SIS).

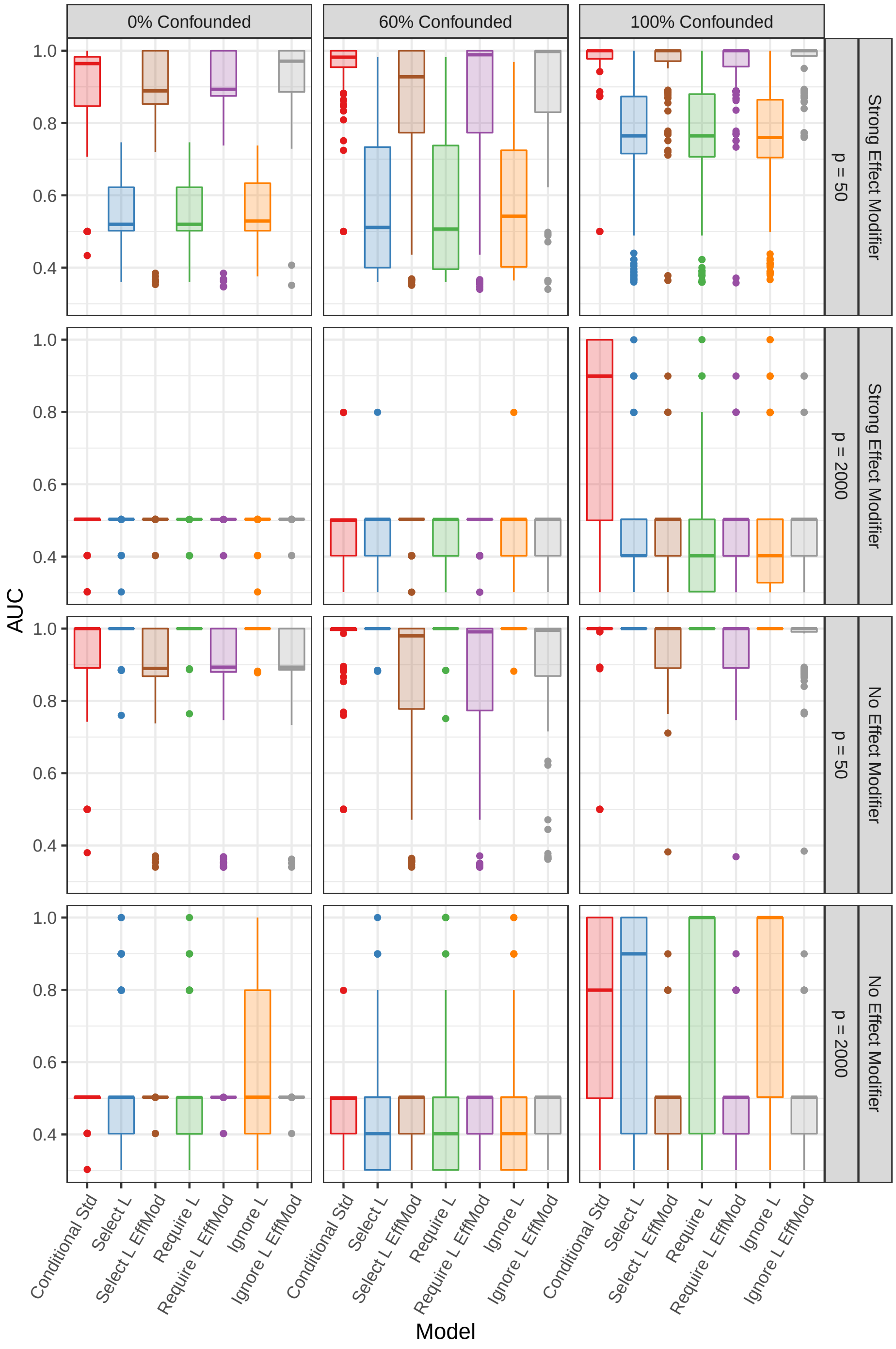

Figure S2: Simulation results: box plots of the area under the curve (AUC) from 100 simulation replications for $n = 50$ and negative binomial features using p-values based on the debiased LASSO estimate following iterative sure independence screening (iterative SIS).

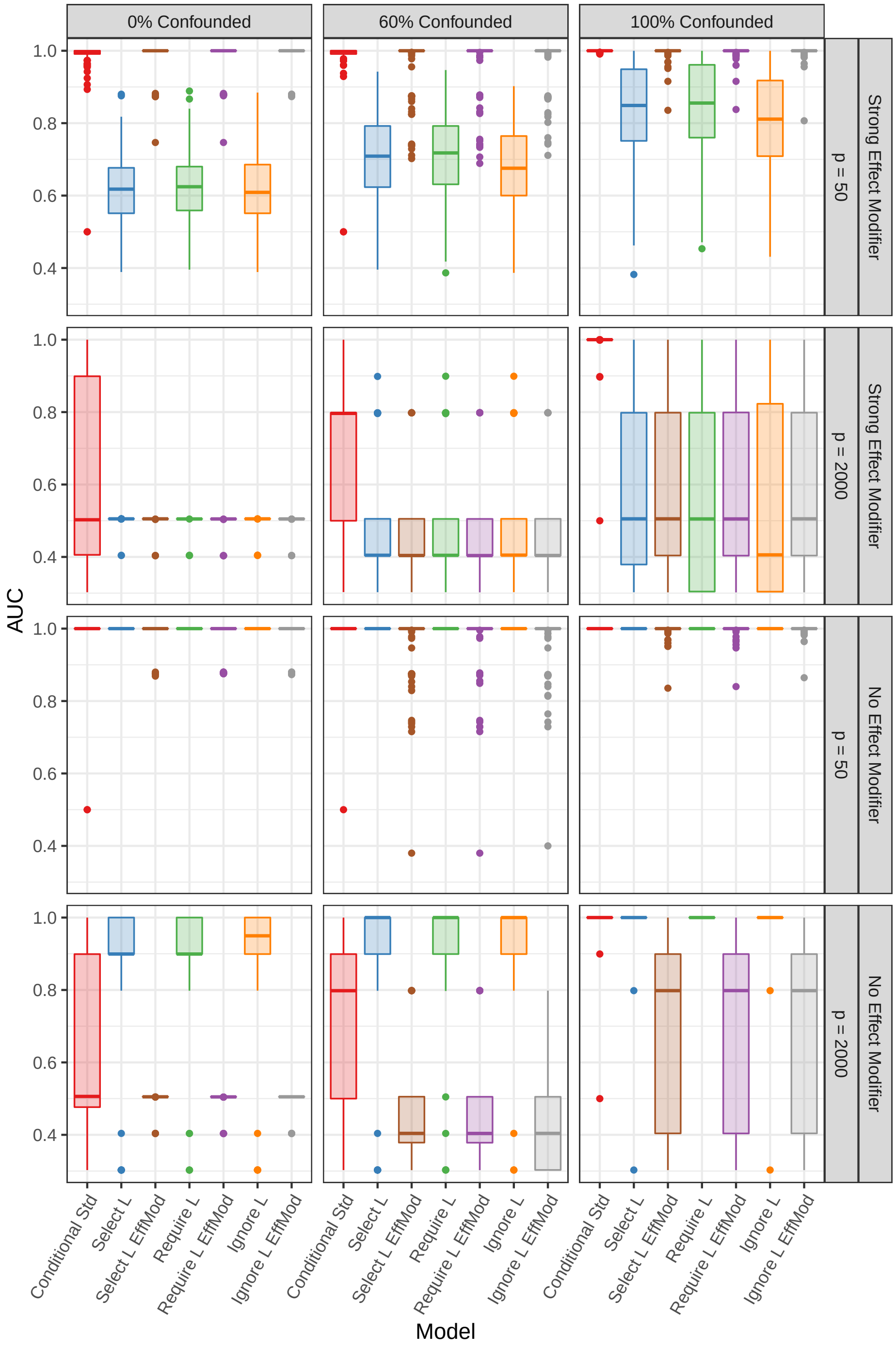

Figure S3: Simulation results: box plots of the area under the curve (AUC) from 100 simulation replications for $n = 100$ and negative binomial features using p-values based on the debiased LASSO estimate following iterative sure independence screening (iterative SIS).

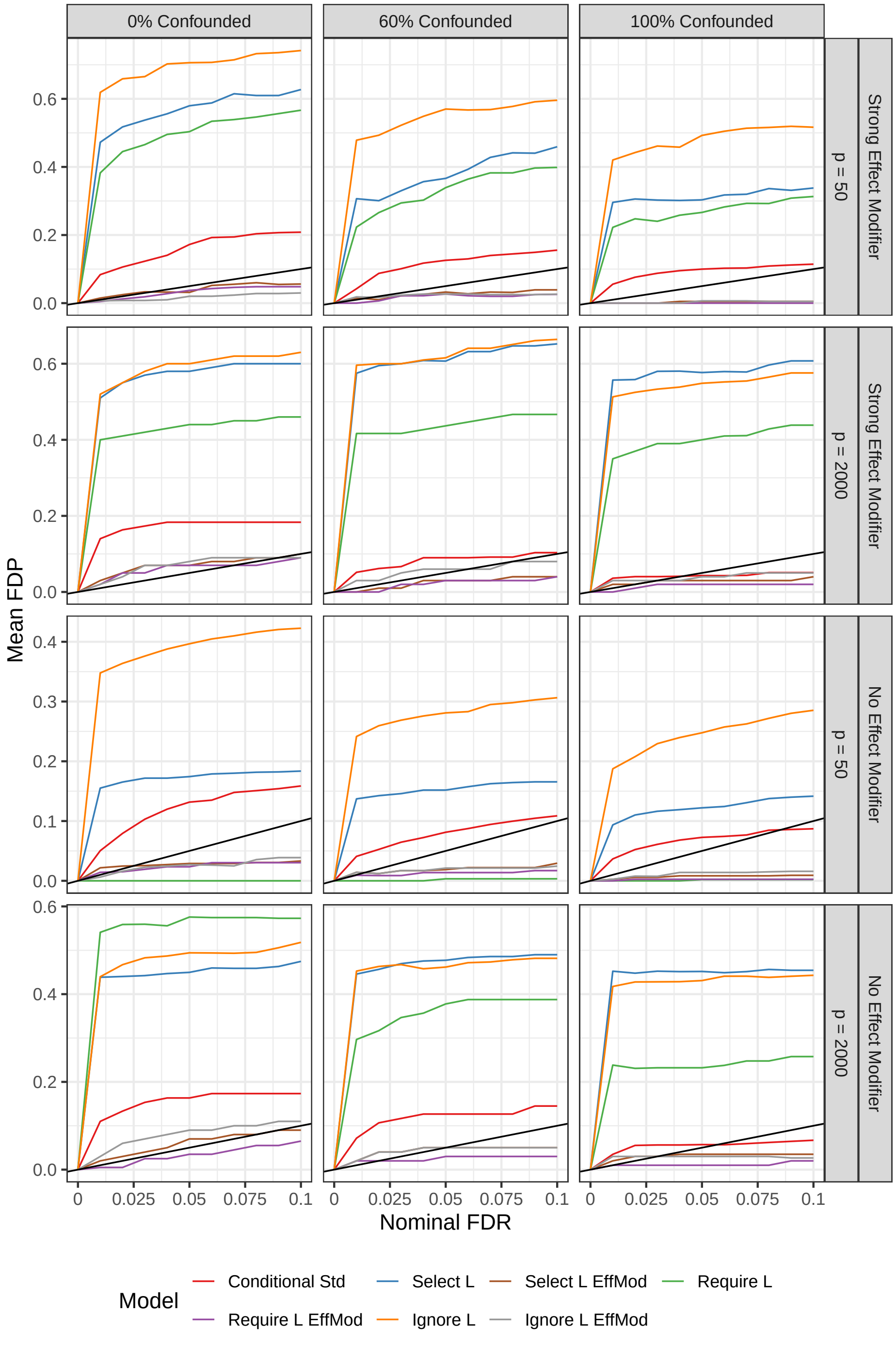

Figure S4: Simulation results: mean estimated false discovery proportion (FDP) for $n = 50$ and Poisson features at varying nominal false discovery rate (FDR) values using Benjamini-Hochberg adjusted p-values based on the debiased LASSO estimate following iterative sure independence screening (iterative SIS). The $y = x$ line is shown in black; any values above this line indicate lack of FDR control.

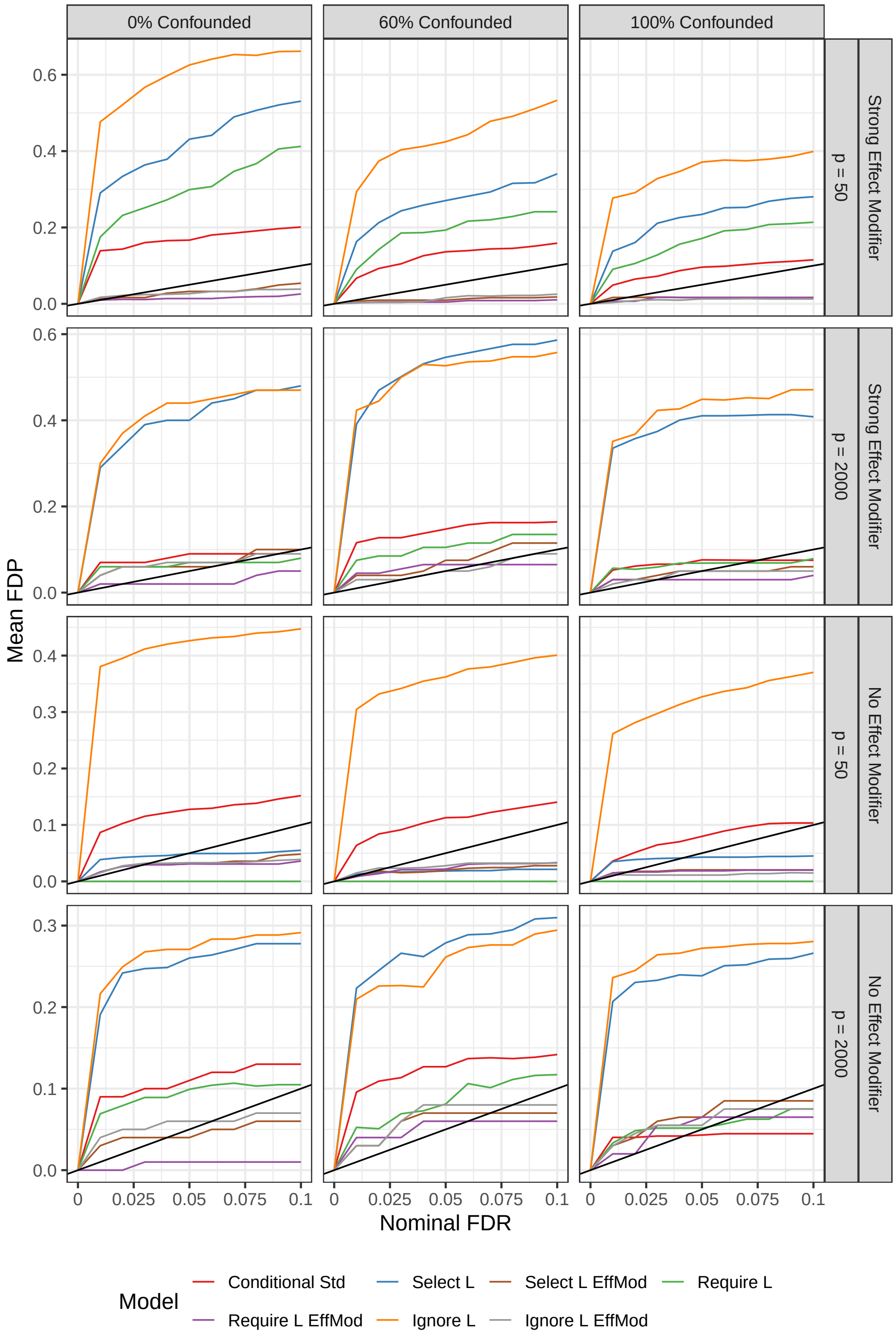

Figure S5: Simulation results: mean estimated false discovery proportion (FDP) for $n = 50$ and negative binomial features at varying nominal false discovery rate (FDR) values using Benjamini-Hochberg adjusted p-values based on the debiased LASSO estimate following iterative sure independence screening (iterative SIS). The $y = x$ line is shown in black; any values above this line indicate lack of FDR control.

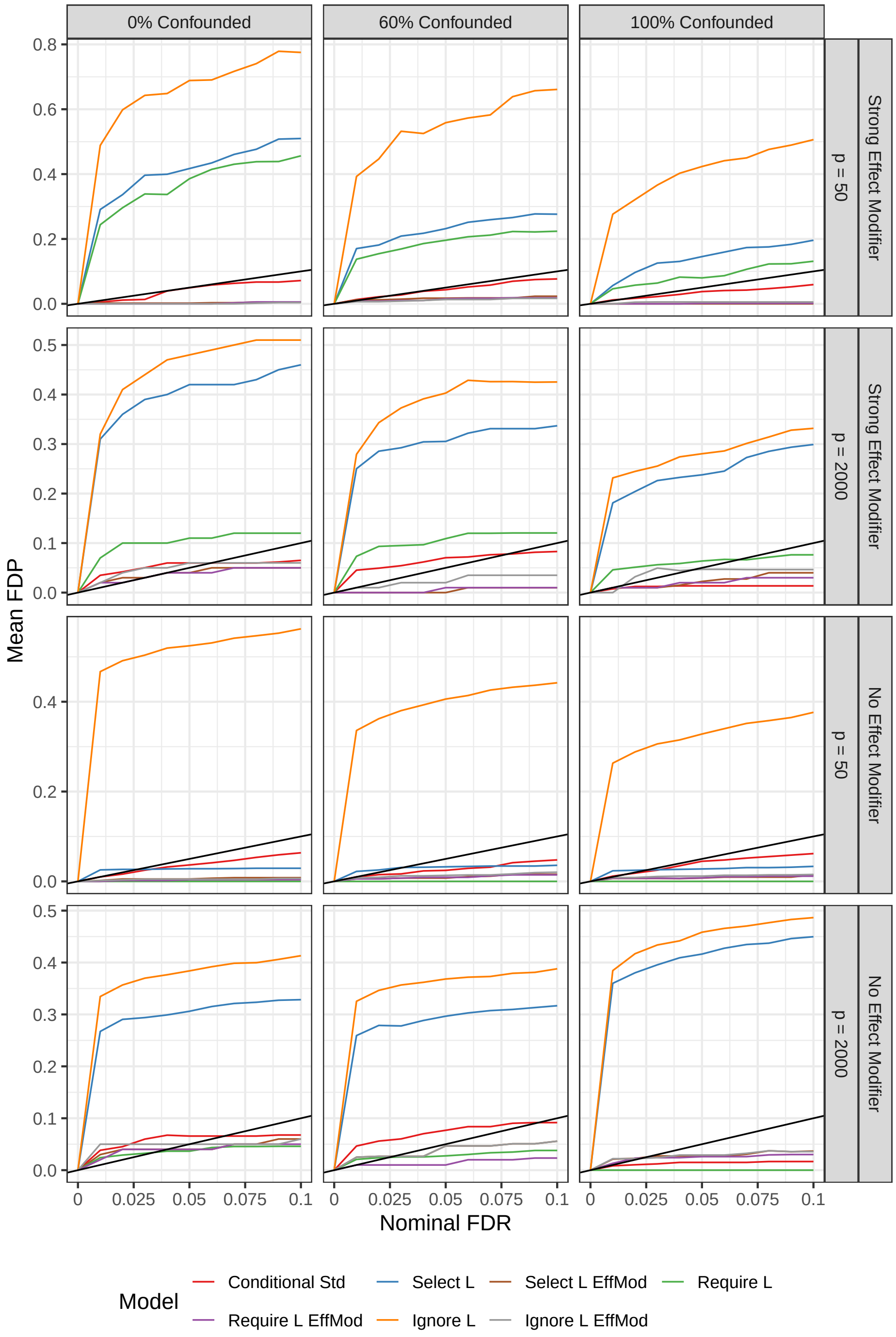

Figure S6: Simulation results: mean estimated false discovery proportion (FDP) for $n = 100$ and negative binomial features at varying nominal false discovery rate (FDR) values using Benjamini-Hochberg adjusted p-values based on the debiased LASSO estimate following iterative sure independence screening (iterative SIS). The $y = x$ line is shown in black; any values above this line indicate lack of FDR control.

S2 Real data analysis: correlation structure and assumption checks

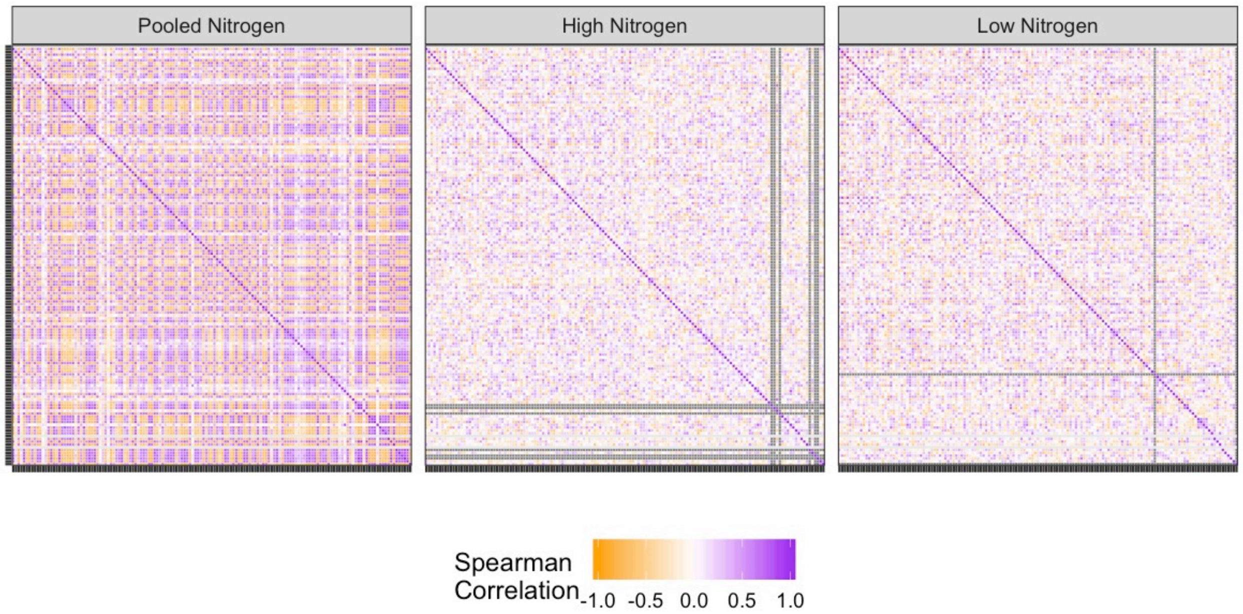

Figure S7: Sorghum microbiome data: Spearman's correlation between the top 150 marginally correlated OTUs for all samples, pooled across both nitrogen conditions (left) and stratified by nitrogen application (center and right).

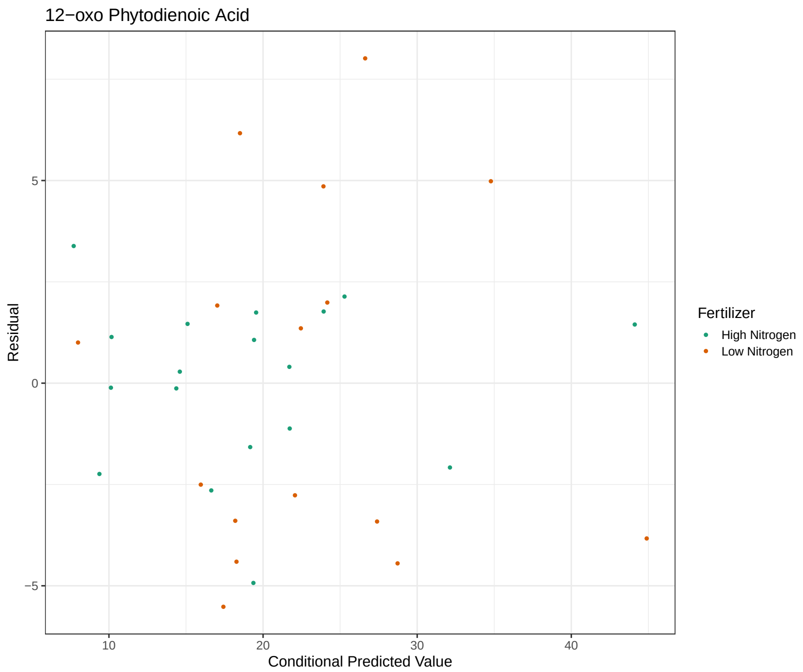

Figure S8: Real data analysis: residuals versus conditional (nitrogen stratum-specific) predicted values from the conditional debiased iterative SIS-LASSO estimates.

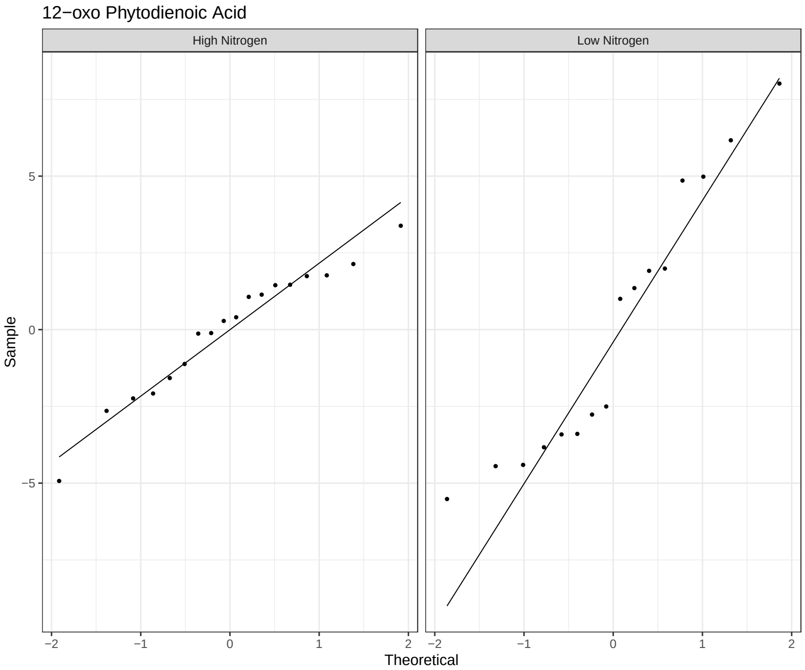

Figure S9: Real data analysis: Q-Q plot of residuals based on conditional (nitrogen stratum-specific) predicted values from the conditional debiased iterative SIS-LASSO estimates.
